# Supplementary material for: mRNA-LNP vaccine encoding the Plasmodium vivax circumsporozoite protein is highly immunogenic and confers protection in mice
Source: Mol Ther Nucleic Acids. 2025 Jul 30;36(3):102645. doi: 10.1016/j.omtn.2025.102645 (PMC12359152; doi:10.1016/j.omtn.2025.102645)
Supplement: Document S1. Figures S1–S3 [file mmc1.pdf]

OMTN, Volume *36*

Supplemental information

mRNA-LNP vaccine encoding the *Plasmodium vivax* circumsporozoite protein is highly immunogenic and confers protection in mice

Amporn Limsalakpetch, Utaiwan Kum-Arb, Kosol Yongvanitchit, Rawiwan Im-Erbsin, Ratawan Ubalee, Norman Waters, Brian A. Vesely, Hiromi Muramatsu, Drew Weissman, Ying K. Tam, Shigeto Yoshida, John Adams, Anjali Yadava, Norbert Pardi, and Sathit Pichyangkul

SUPPLEMENTAL MATERIAL

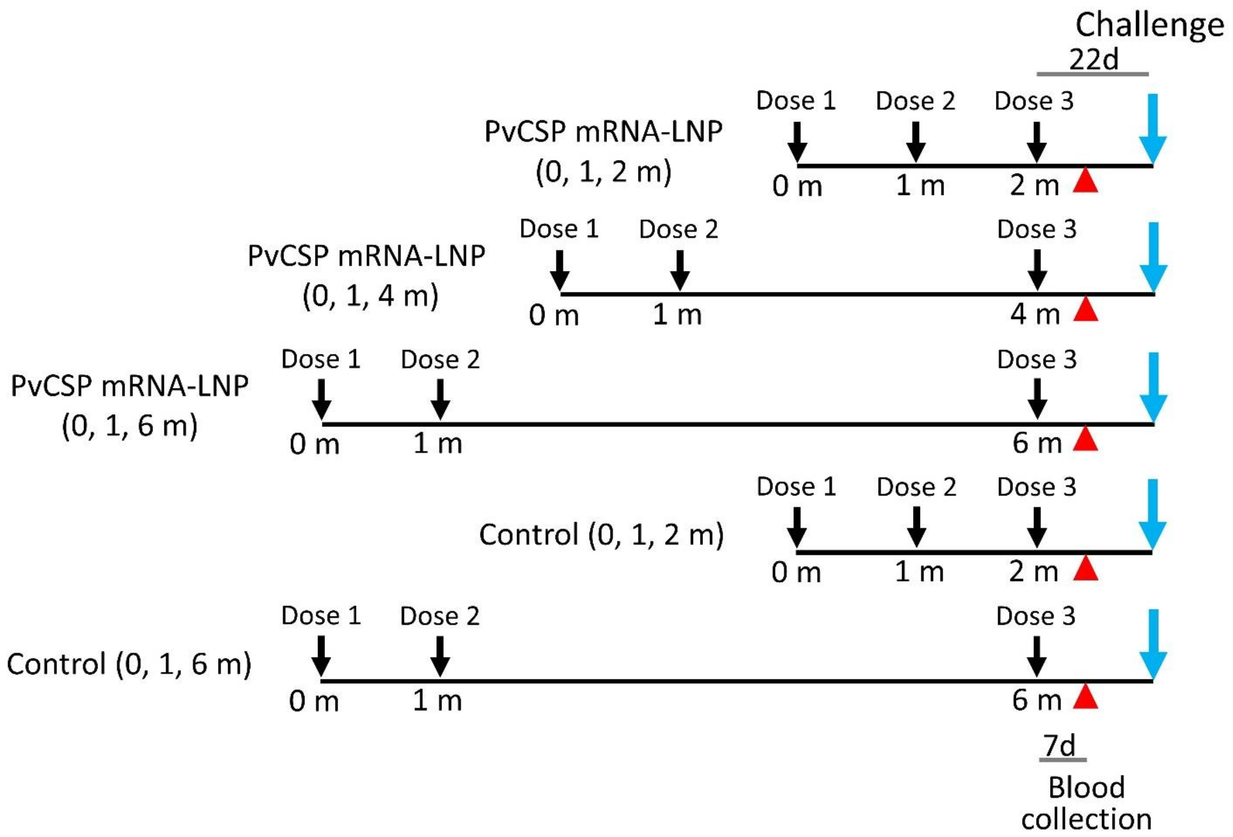

Figure S1. Vaccination regimen for testing the protective efficacy of the PvCSP mRNA-LNP vaccine

Mice (n = 10 per group) were IM immunized with 3 doses of the PvCSP mRNA-LNP vaccine (30 µg) at either 0, 1, and 2 months; 0, 1, and 4 months; or 0, 1, and 6 months. Two separate control groups received poly(C) RNA-LNP (30 µg per dose) at 0, 1, and 2 months, or at 0, 1, and 6 months. Serum samples were collected seven days after the final vaccine dose to measure antibody responses. All mice were challenged with transgenic sporozoites twenty two days after the last vaccine dose.

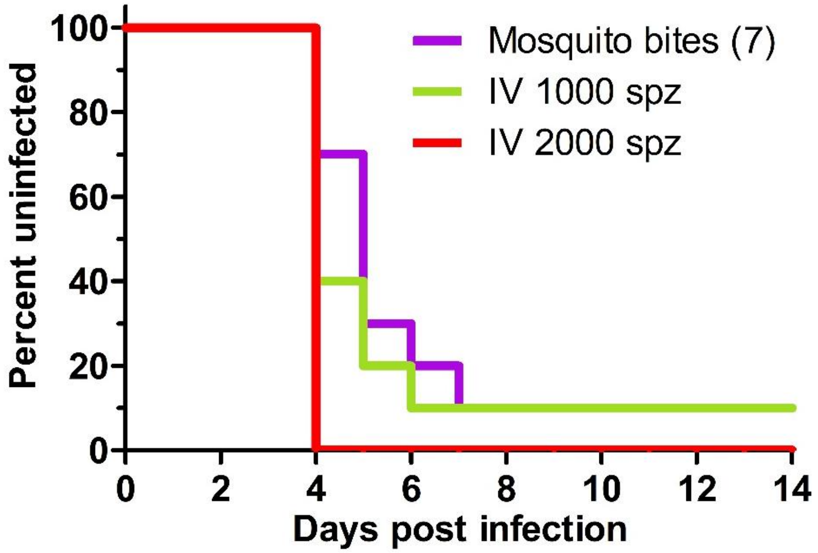

Figure S2. Transgenic P. berghei sporozoites expressing PvCSP (VK210) effectively establish a blood stage parasitemia in CD-1 outbred mice

In the study, mice (n=10 per group) were exposed to seven mosquito bites, or administered 1,000 or 2,000 sporozoites intravenously (IV). Administration of 2,000 sporozoites resulted in a 100% incidence of blood stage infection.

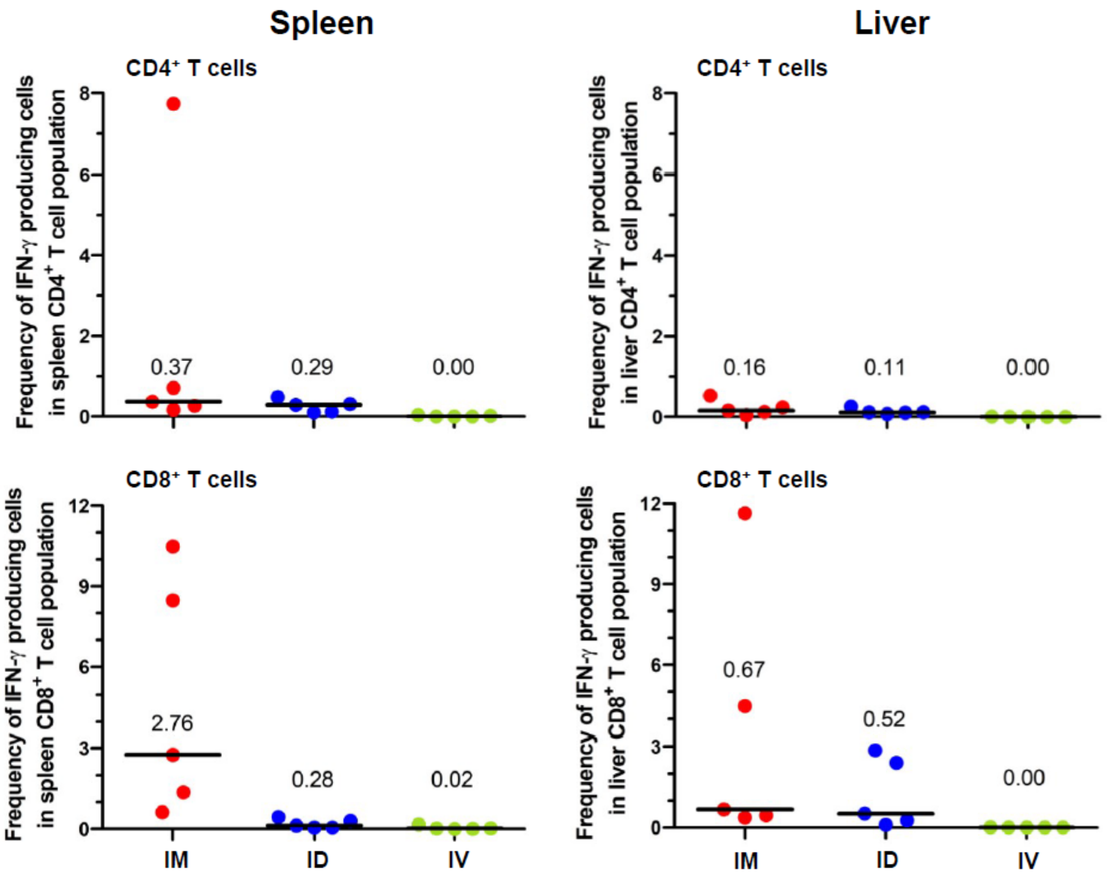

Figure S3. IV administration of the PvCSP mRNA-LNP vaccine induces a minimal T cell response compared to the IM and ID administration routes

Mice (n=5 per group) were immunized with the PvCSP mRNA-LNP vaccine (30 µg/dose) via IM, ID, or IV routes at 0 and 4 weeks. CD4^{+} and CD8^{+} memory T cell responses in the spleen and liver were measured for cytokine production 29-41 days after the final vaccination using an ICS assay. Each dot represents one animal, and horizontal lines indicate the medians.
