# Supplementary material for: mRNA-LNP vaccine encoding the Plasmodium vivax circumsporozoite protein is highly immunogenic and confers protection in mice
Source: Mol Ther Nucleic Acids. 2025 Jul 30;36(3):102645. doi: 10.1016/j.omtn.2025.102645 (PMC12359152; doi:10.1016/j.omtn.2025.102645)
Supplement: Document S2. Article plus supplemental information [file mmc2.pdf]

Molecular Therapy
Nucleic Acids
Original Article

mRNA-LNP vaccine encoding the *Plasmodium vivax* circumsporozoite protein is highly immunogenic and confers protection in mice

Amporn Limsalakpetch,[1,8] Utaiwan Kum-Arb,[1,8] Kosol Yongvanitchit,[1] Rawiwan Im-Erbsin,[1] Ratawan Ubalee,[1] Norman Waters,[1,9] Brian A. Vesely,[1] Hiromi Muramatsu,[2] Drew Weissman,[3] Ying K. Tam,[4] Shigeto Yoshida,[5] John Adams,[6] Anjali Yadava,[7] Norbert Pardi,[2] and Sathit Pichyangkul[1]

[1]Department of Bacterial and Parasitic Diseases, WRAIR-AFRIMS, Bangkok 10400, Thailand; [2]Department of Microbiology, Perelman School of Medicine, University of Pennsylvania, Philadelphia, PA 19104, USA; [3]Department of Medicine, Perelman School of Medicine, University of Pennsylvania, Philadelphia, PA 19104, USA; [4]Acuitas Therapeutics, Vancouver, BC V6T 1Z3, Canada; [5]Laboratory of Vaccinology and Applied Immunology, School of Pharmacy, Kanazawa University, Kanazawa, Ishikawa 920-1192, Japan; [6]Center for Global Health and Infectious Diseases Research and USF Genomics Program, College of Public Health, University of South Florida, Tampa, FL 33612, USA; [7]Biologics Research & Development Branch, CIDR, Walter Reed Army Institute of Research, Silver Spring, MD 20910, USA

***Plasmodium vivax* poses significant challenges to malaria control due to its relapsing nature. This study explores the immunogenicity and efficacy of nucleoside-modified mRNA-lipid nanoparticle (LNP) vaccines targeting the *P. vivax* circumsporozoite protein (PvCSP). Two mRNA constructs encoding PvCSP were designed and tested in mice. Despite lower protein expression, the vaccine encoding the wild-type signal peptide (SP) and glycosylphosphatidylinositol (GPI) anchor of PvCSP induced significantly higher antibody titers against the PvCSP and its repeat region compared with the mRNA construct with SP but without GPI. The immunogenicity of PvCSP mRNA-LNP vaccines was evaluated using various administration routes and immunization schedules. Both intradermal and intramuscular delivery generated dose-dependent antibody responses, but the former demonstrated superior responses at a lower dose. Conversely, intravenous administration resulted in very poor responses. Notably, administering a delayed third dose intramuscularly 5 months after the second dose resulted in significantly higher levels of anti-repeat region antibodies and enhanced T cell responses in both the spleen and liver. This delayed regimen provided strong protection against sporozoite challenge, with the magnitude and avidity of anti-repeat region antibodies linked to this protection. These findings highlight the potential of the nucleoside-modified mRNA-LNP vaccine platform in combating *P. vivax* pre-erythrocytic stage infection.**

INTRODUCTION

Despite sustained efforts in disease control over recent decades, malaria remains a significant global public health challenge, particularly in many low- and middle-income countries.[1] The World Malaria Report 2023 documented 249 million malaria cases in 2022, resulting in 608,000 deaths, predominantly affecting children in sub-Saharan Africa.[2] Among human malaria infections, the primary causative agents are *Plasmodium falciparum* and *Plasmodium vivax*.[3] The majority of malaria-related deaths in sub-Saharan Africa are caused by *P. falciparum*, whereas *P. vivax* exhibits a broad geographical distribution[4] and is now responsible for most malaria cases in the Asia-Pacific and South America regions, where it is associated with severe disease outcomes.[5]

Unlike *P. falciparum*, *P. vivax* possesses unique traits that complicate its control and enable its persistence. These traits include the ability to develop in a range of mosquito vectors at lower temperatures, causing low-density infections that are often undetectable by routine tests, and early production of gametocytes, which facilitates transmission before symptoms appear.[6] Most notably, *P. vivax* can form dormant liver stages (hypnozoites) that lead to frequent relapses, posing challenges for treatment and control efforts.[7] Current treatment options, such as primaquine and tafenoquine, are limited by serious side effects, including acute hemolysis in individuals with glucose-6-phosphate dehydrogenase deficiency,[8] a common enzyme deficiency that affects approximately 400 million people worldwide.[9] Additionally, these drugs cannot be administered during pregnancy or to young children under 6 months of age.[10]

Vaccination represents the most cost-effective approach to controlling and eliminating *P. vivax* malaria. However, the development of *P. vivax* vaccines has lagged behind vaccine development against *P. falciparum*. While the World Health Organization recently endorsed

Received 30 January 2025; accepted 25 July 2025;
https://doi.org/10.1016/j.omtn.2025.102645.

[8]These authors contributed equally

[9]Present address: Sabin Vaccine Institute, Washington, DC 20006, USA

Correspondence: Sathit Pichyangkul, Immunology and Vaccinology Section, Department of Bacterial and Parasitic Diseases, WRAIR-AFRIMS, Bangkok, Thailand.
E-mail: sathitp@afrims.org

Published by Elsevier Inc. on behalf of The American Society of Gene and Cell Therapy.
This is an open access article under the CC BY license (http://creativecommons.org/licenses/by/4.0/).

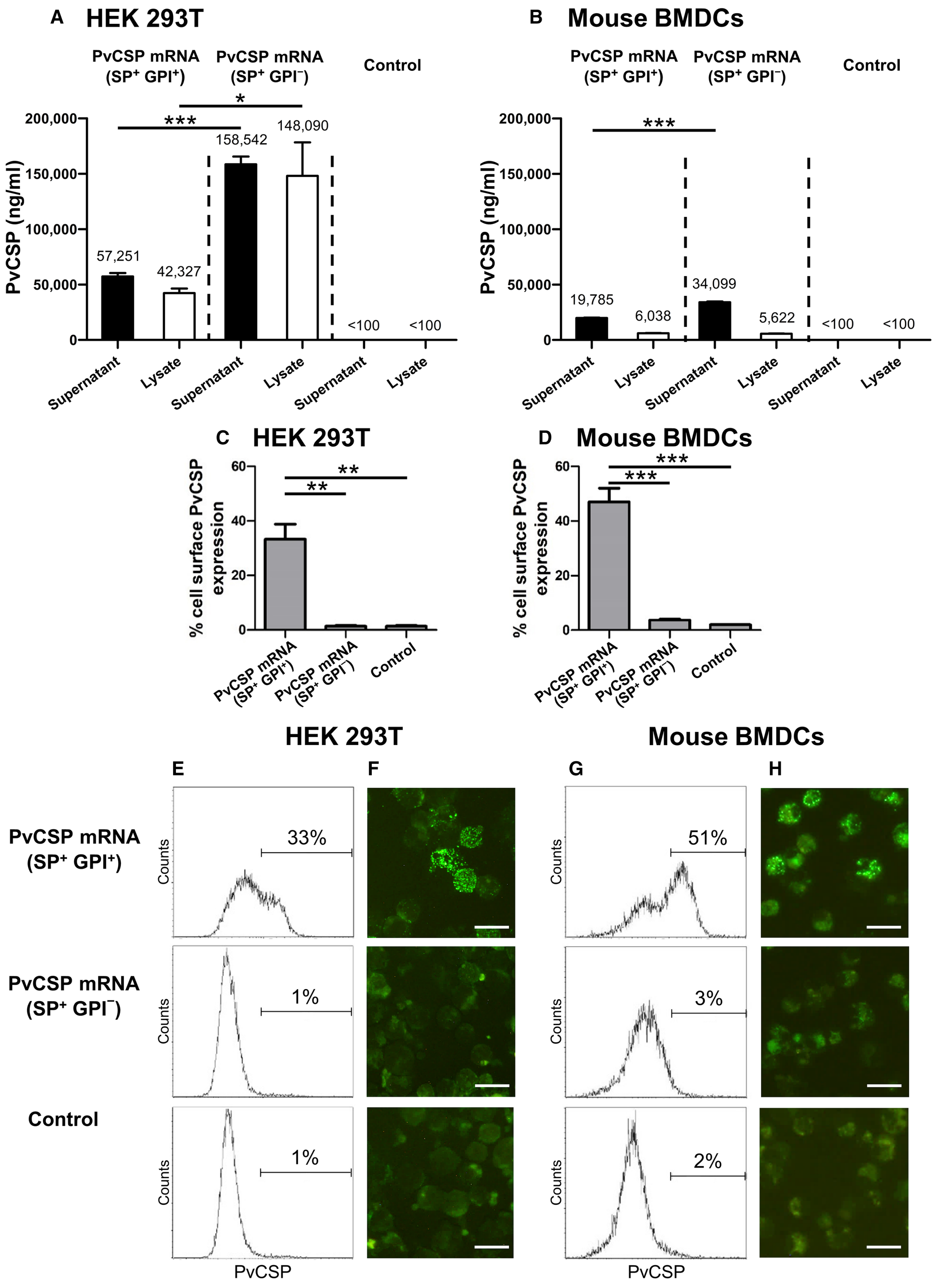

(legend on next page)

two vaccines, the RTS,S/AS01 and R21/Matrix-M vaccines,[11,12] against *P. falciparum* malaria, few candidates targeting *P. vivax* have progressed to clinical trials. The initial pre-erythrocytic stage vaccine based on *P. vivax* circumsporozoite protein (PvCSP), known as VMP001/AS01B, was developed by the Walter Reed Army Institute of Research (WRAIR), demonstrated safety and delayed parasitemia but did not achieve sterile protection in clinical trials in the United States.[13] A recent phase II trial of a PvCSP vaccine, using three long synthetic peptides covering different regions of PvCSP and formulated with Montanide ISA-51 adjuvant, demonstrated moderate efficacy (approximately 54%) in controlled human malaria infection (CHMI).[14] Further studies with larger participant pools are ongoing to confirm these findings. Thus, there remains an urgent need for a new and highly immunogenic vaccine platform for PvCSP-based vaccines.

Inspired by the groundbreaking success of nucleoside-modified mRNA-lipid nanoparticle (LNP) vaccines against severe acute respiratory syndrome coronavirus 2, which have proven safe and highly effective, saving millions of lives during the recent pandemic,[15–17] there has been a surge in exploring this platform for developing vaccines against other infectious diseases, especially those currently without vaccines. In this preliminary proof-of-concept study, we investigated the potential of mRNA-LNP as a platform for a viable PvCSP vaccine. We found that vaccination with mRNA-LNP encoding PvCSP (VK210) elicited robust antibody and T cell responses. Importantly, it conferred a high level of protection in mice after challenge with transgenic sporozoites expressing PvCSP. These results support the promise of mRNA-LNP-based vaccines for *P. vivax* malaria.

RESULTS

Design of two mRNA constructs encoding PvCSP and *in vitro* protein expression

The N1-methylpseudouridine (m1Ψ)-modified PvCSP mRNAs were designed based on the sequence of the PvCSP (VK210) gene from the reference *P. vivax* strain Salvador 1. Two PvCSP mRNA constructs were synthesized. Construct 1 included the wild-type signal peptide (SP) along with the glycosylphosphatidylinositol (GPI) anchor (SP^+ GPI^+), while construct 2 featured the wild-type SP but lacked the GPI anchor (SP^+ $GPI^−$). The expression of the PvCSP protein from the encoding mRNA was validated by transfecting human embryonic kidney (HEK) 293T cells and measuring PvCSP expression 18 h later by ELISA. mRNA construct 2 (SP^+ $GPI^−$) showed significantly higher levels of PvCSP protein in the cell culture supernatants and cell lysates compared with mRNA construct 1 (SP^+ GPI^+) (Figure 1A).

Subsequently, we evaluated PvCSP expression in primary murine bone marrow-derived dendritic cells (BMDCs). BMDCs transfected with mRNA construct 2 (SP^+ $GPI^−$) also produced significantly higher levels of PvCSP in the cell culture supernatants compared with construct 1. However, there was no significant difference in PvCSP levels in the cell lysates when compared with those transfected with mRNA construct 1 (SP^+ GPI^+) (Figure 1B).

In addition to measuring PvCSP levels in the culture supernatants and cell lysates, we also evaluated its expression on the cell surface of HEK 293T cells and BMDCs. Our results demonstrated that transfecting cells with mRNA construct 1 (SP^+ GPI^+) for 15–18 h significantly increased the expression of PvCSP on the surface of transfected cells. Specifically, 33 ± 5.5% (mean ± SEM) of HEK 293T cells (Figure 1C) and 47 ± 5.0% of BMDCs ($CD11c^+$ MHC class II^+) (Figure 1D) exhibited positive cell-surface expression. In contrast, transfection with mRNA construct 2 (SP^+ $GPI^−$) and the control resulted in minimal expression of the PvCSP protein on the cell surface. Specifically, only 1 ± 0.3% of HEK 293T cells and 4 ± 0.3% and 2 ± 0% of BMDCs, respectively, showed positive expression (Figures 1C and 1D). Figures 1E and 1F show representative flow cytometry histograms and stained cells under a fluorescent microscope for HEK 293T cells, while Figures 1G and 1H display the same for BMDCs.

PvCSP mRNA-LNP vaccine containing SP^+ GPI^+ induces a robust antibody response

Since the two mRNA constructs encoding PvCSP exhibited varying levels of protein and cell-surface expression, especially in mouse BMDCs, their ability to elicit an immune response was assessed. Both mRNA constructs were encapsulated in LNPs and the vaccines were evaluated in CD-1 outbred mice. The immune responses of CD-1 outbred mice are more representative of the diversity observed in outbred human populations compared with those of inbred mouse strains.[18,19] This outbred mouse strain was used throughout the study.

A two-dose vaccine regimen administered 4 weeks apart was used for testing. mRNA construct 1 [PvCSP (SP^+ GPI^+)], showing lower

Figure 1. *In vitro* expression of mRNA-encoded PvCSP protein after transfection of HEK 293T cells and mouse BMDCs

Two versions of PvCSP mRNA were transfected into HEK 293T cells and mouse BMDCs using Lipofectamine 2000: one with the wild-type SP and GPI anchor (SP^+ GPI^+) and the other with the wild-type SP but with no GPI (SP^+ $GPI^−$). Cells treated with Lipofectamine 2000 (no mRNA) were used as non-transfected controls. The presence of translated PvCSP protein in the culture supernatants and cell lysates of both transfected and non-transfected HEK 293T cells (A) and BMDCs (B) was measured using an ELISA specific to PvCSP (VK210). The data shown are the mean ± SEM of four independent experiments. Statistical significance was determined by a paired t test.*$p < 0.05$, ***$p < 0.001$. Cell-surface expression of PvCSP at 15–18 h post transfection in HEK 293T cells (C) and BMDCs (D) was assessed by flow cytometry. Data are shown as mean ± SEM of three independent experiments. Statistical significance was determined by one-way ANOVA with Tukey's post-test. **$p < 0.01$, ***$p < 0.001$. Representative flow cytometry histogram of three independent experiments showing cell-surface expression of PvCSP in HEK 293T cells (E) and BMDCs (G). Stained HEK 293T cells (F) and BMDCs (H) were analyzed under fluorescent microscope.
Scale bars, 20 μm.

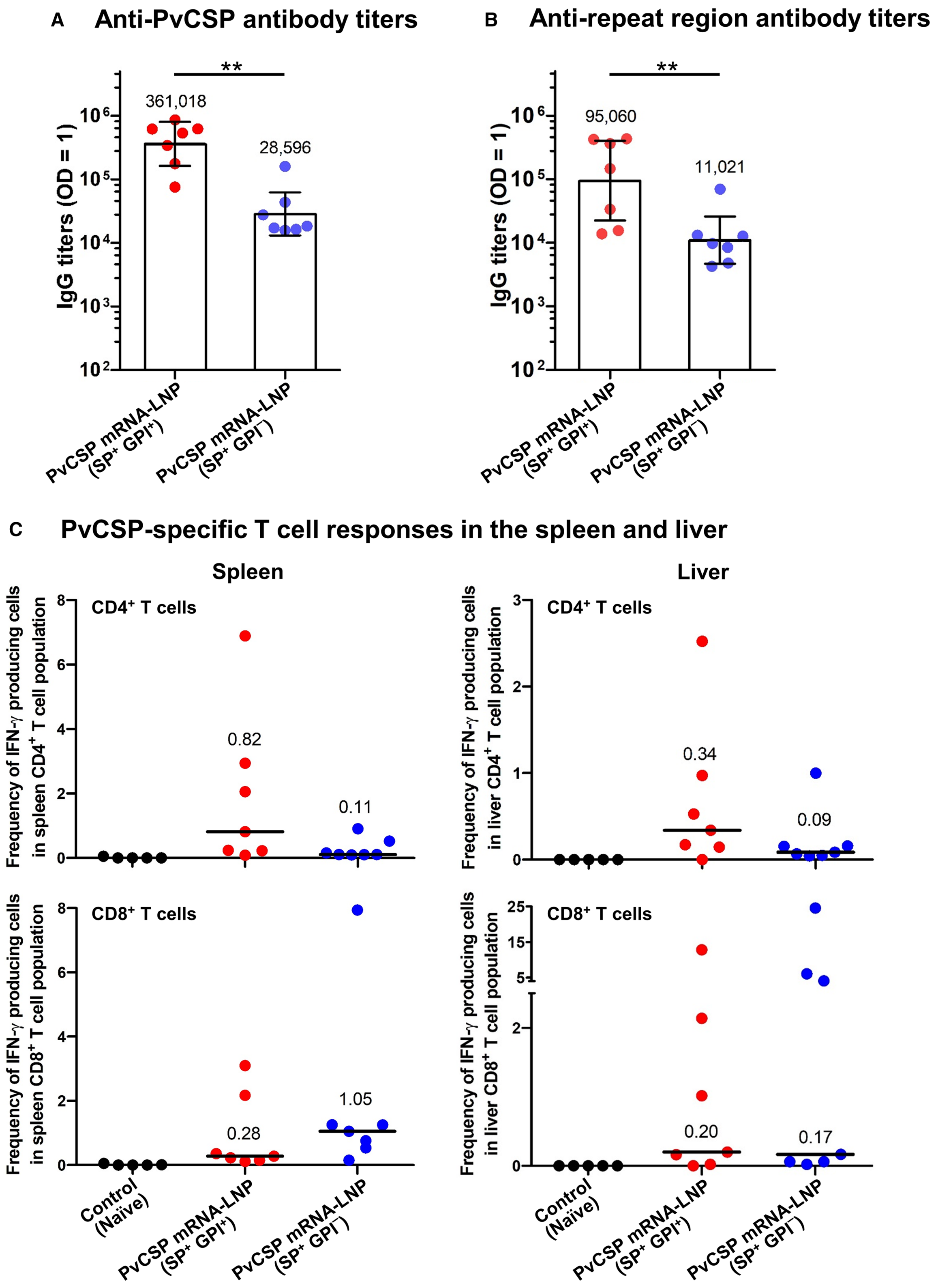

(legend on next page)

overall protein expression but high cell-surface expression, elicited a significantly stronger antibody response compared with construct 2, which encoded PvCSP (SP^+ GPI^-). One week after administration of the second dose, mRNA construct 1 induced antibody responses that were 13 times greater against the PvCSP and 9 times greater against the repeat region compared with construct 2 (Figures 2A and 2B).

While both mRNA-LNPs successfully triggered T cell responses in the spleen and liver, the differences in interferon (IFN)-γ^+ $CD4^+$ and IFN-γ^+ $CD8^+$ T cell responses between the two constructs were not significant (Figure 2C). Consequently, the PvCSP (SP^+ GPI^+) mRNA-LNP vaccine was selected for subsequent experiments.

Assessing the antibody response elicited by the PvCSP (SP^+ GPI^+) mRNA-LNP vaccine through different routes of administration

The immunogenicity of vaccines can be influenced by the route of administration.[20,21] We examined the antibody response after administering the PvCSP mRNA-LNP vaccine via the intramuscular (IM), intradermal (ID), and intravenous (i.v.) routes, with doses of 10 and 30 μg administered 4 weeks apart. When administering the vaccine via either the IM or ID routes, we observed a dose-dependent antibody response specific to both the PvCSP (Figure 3A) and the repeat region (Figure 3B). It is worth noting that the i.v. administration of the vaccine resulted in a significantly poorer antibody response against the PvCSP and the repeat region compared with the IM and ID administrations (Figures 3A and 3B).

Generally, animals that received the vaccine via ID administration showed an increase in their antibody response against both the PvCSP and the repeat region compared with the IM-injected mice (Figures 3A and 3B). IM administration of the PvCSP mRNA vaccine at a dose of 30 μg elicited significantly higher antibody levels against both PvCSP and the repeat region compared with the 10-μg dose (Figures 3A and 3B).

Immunofluorescence assay (IFA) results demonstrate that antibodies induced by the PvCSP mRNA-LNP vaccine were able to recognize the native CSP expressed on *P. vivax* sporozoites (VK210) isolated from the salivary glands of infected mosquitoes, as well as transgenic *P. berghei* sporozoites expressing PvCSP (VK210) (Figure 3C).

Immunogenicity of a three-dose IM vaccine regimen

A recent study revealed that a three-dose regimen of the IM-administered mRNA-LNP vaccine encoding *P. falciparum* CSP offers greater protection than a two-dose regimen.[22] Thus, in this experiment, we aimed to investigate whether administering a delayed third dose either 3 or 5 months after administration of the second dose could enhance antibody and T cell responses compared with a schedule of three vaccinations given monthly at 0, 1, and 2 months. A 30-μg dose of the PvCSP mRNA vaccine was selected, as it significantly induced a higher antibody response than the 10-μg dose.

All three vaccination schedules produced strong antibody responses 2 weeks after the final vaccination. There was no significant difference in the antibody response against the PvCSP among these schedules (Figure 4A). Antibody responses specific to the N-terminal and C-terminal regions of PvCSP were also evaluated, and no significant differences were found (Figures 4B and 4C). However, a significant increase in antibodies specific to the repeat region was observed with a delayed third dose administered 3 or 5 months after the second dose (Figure 4D). We next evaluated the immunoglobulin (Ig)G isotype profiles by assessing the ratio of IgG2a to IgG1 of anti-repeat region antibodies and their avidity. There was no significant difference in the ratio of IgG2a to IgG1 antibodies targeting the repeat region across the three vaccination schedules (Figure 4E). There was a small increase in the anti-repeat region antibody avidity in animals receiving the delayed third dose, but the difference was not significant (Figure 4F).

PvCSP-specific $CD4^+$ and $CD8^+$ memory T cell responses were detected in both the spleen and liver 4 weeks after administration of the last vaccine dose. Notably, animals that received a delayed third dose, particularly those that received the third dose 5 months after the second dose, showed elevated IFN-γ^+ $CD4^+$ and IFN-γ^+ $CD8^+$ T cell responses (Figure 4G). This group exhibited significantly higher frequencies of $CD4^+$ T cells secreting IFN-γ in both the spleen and liver compared with animals vaccinated at 0, 1, and 2 months. Furthermore, animals vaccinated at 0, 1, and 4 months also displayed a trend toward increased IFN-γ^+ $CD4^+$ T cell responses in the spleen and liver, although this trend did not attain statistical significance when compared with vaccination at 0, 1, and 2 months.

In terms of the $CD8^+$ memory T cell response, the frequencies of $CD8^+$ T cells secreting IFN-γ in the spleen of animals vaccinated at 0, 1, and 6 months were significantly higher than those vaccinated at 0, 1, and 4 months and 0, 1, and 2 months (Figure 4G). Additionally, although not statistically significant, there was a trend toward increased IFN-γ^+ $CD8^+$ T cell responses in the liver of animals vaccinated at 0, 1, and 6 months.

Figure 2. PvCSP mRNA (SP^+ GPI^+)-LNP vaccine elicits a superior antibody response

Mice ($n = 7$ per group) were IM immunized with 30 μg of PvCSP mRNA (SP^+ GPI^+)-LNP or PvCSP mRNA (SP^+ GPI^-)-LNP vaccines. One week after administration of the last vaccine dose, serum samples from immunized mice were assessed for binding antibodies specific to the PvCSP (A) and the repeat region (B). Antibody titers detected by ELISA were defined as the dilution required to achieve an OD of 1 at 405 nm. Each dot represents one animal. Data in the bar graphs are presented as geometric mean titers and 95% confidence intervals. Statistical significance was determined by a Mann-Whitney U test on log-transformed values. $^{**}p < 0.01$. $CD4^+$ and $CD8^+$ T cell responses in the spleen and liver were measured for IFN-γ production using an ICS assay 29–41 days after the second immunization (C). $CD4^+$ and $CD8^+$ T cell responses in the spleen and liver of naive mice were used as controls ($n = 5$). Each dot represents one animal, and horizontal lines indicate the medians.

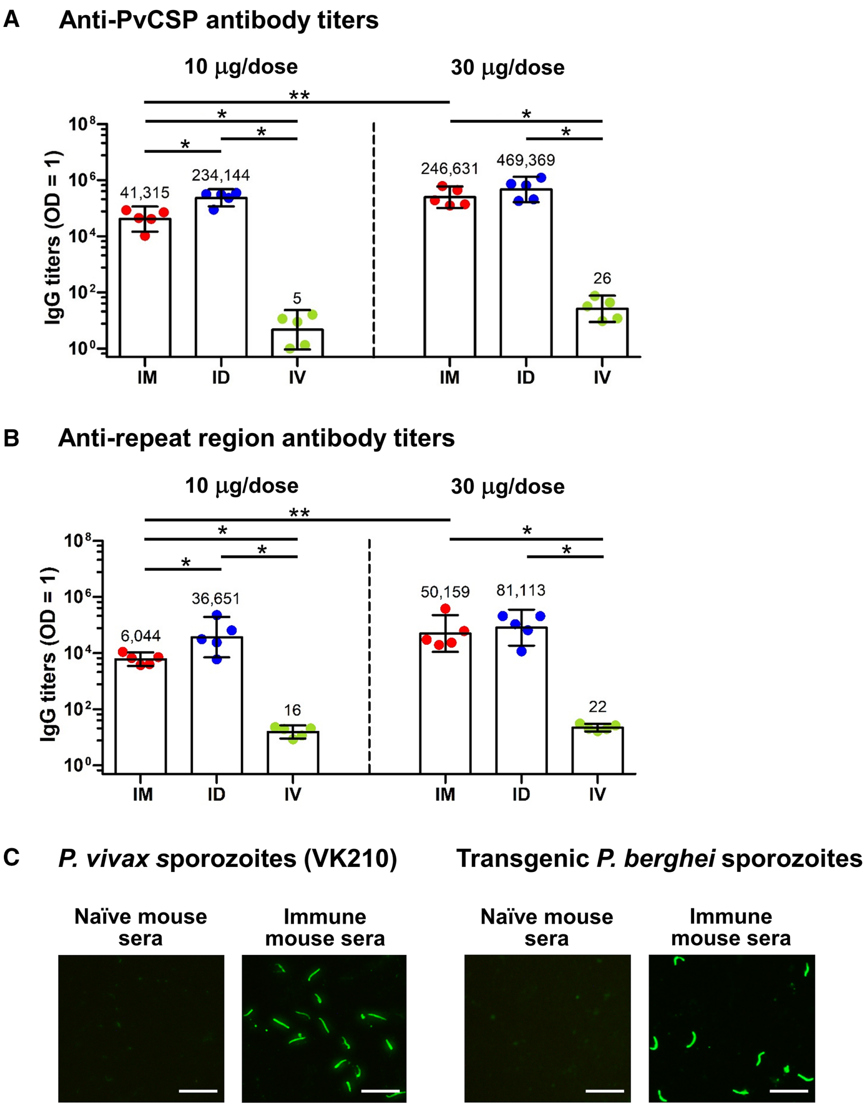

Figure 3. Antibody response generated by the PvCSP mRNA-LNP vaccine when administered via IM, ID, or i.v. routes

Mice (n = 5 per group) were immunized twice with 10 or 30 μg doses of the PvCSP mRNA-LNP vaccine via IM, ID, or i.v. routes. One week after administration of the last vaccine dose, serum samples from the immunized mice were assessed for antibodies specific to the PvCSP (A) and the repeat region (B). Each dot represents one animal. Data in the bar graphs are shown as geometric mean titers and 95% confidence intervals. Statistical significance was determined by one-way ANOVA with Tukey's post test or a Mann-Whitney U test on log-transform values. Pooled serum antibodies collected 1 week after administration of the second dose (30 μg/dose, IM) and diluted at 1:10^5 recognized CSP expressed on *P. vivax* sporozoites (VK210) and transgenic *P. berghei* sporozoites expressing PvCSP (VK210), as assessed by IFA (C). Serum pooled from naive mice (1:10^2 dilution) showed no reactivity against PvCSP.
Scale bars, 20 μm. *$p < 0.05$, **$p < 0.01$.

A delayed third dose given 5 months after administration of the second dose elicits superior protection in a sporozoite challenge mouse model

To evaluate the protective efficacy of the PvCSP mRNA-LNP vaccine, animals were immunized IM with a 30-μg dose using a three-dose vaccination regimen (Figure S1). Twenty-two days after administration of the last vaccine dose, they were challenged with an i.v. injection of 2,000 transgenic *P. berghei* sporozoites expressing PvCSP (VK210). This sporozoite dose has been shown to result in a 100% blood stage infection in CD-1 outbred mice (Figure S2). Although the mosquito bite is the natural mode of malaria infection, the i.v. challenge represents a much more stringent model. Every control animal that received polycytidylic acid (poly(C)) RNA-LNP-negative control either at 0, 1, and 2 months or at 0, 1, and 6 months became positive for blood stage infection by day 5 after sporozoite challenge. In contrast, 7 of 10 mice vaccinated with PvCSP mRNA-LNP at 0, 1, and 2 months were protected, and 8 of 10 mice vaccinated at 0, 1, and 4 months also showed protection. The highest level of protection (9 of 10) was observed in animals vaccinated at 0, 1, and 6 months, demonstrating 90% sterile protection compared with control animals receiving poly(C) RNA-LNP (Figure 5A).

To further explore the association between the magnitude of antibody responses and protection, we collected blood 15 days before the sporozoite challenge and evaluated antibody titers specific to the PvCSP and the repeat region. Our analyses revealed no association between protection and antibody titers against the PvCSP (Figure 5B). However, we observed a significant correlation between antibody titers against the repeat region and protection (Figure 5C). We also assessed the antibody response to a specific tetramer, AGDR, located in the repeat region known to be targeted by neutralizing antibodies.[23,24] The vaccine-induced antibody response against AGDR was modest, and we did not observe any association between AGDR-specific antibody levels and protection (Figure 5D).

Furthermore, the ratio of IgG2a to IgG1 antibodies targeting the repeat region was not associated with protection (Figure 5E). However, the IgG avidity index to the repeat region significantly correlated with protection (Figure 5F).

DISCUSSION

Several mRNA-LNP vaccines targeting *P. falciparum* antigens, including PfCSP and transmission-blocking antigens (Pfs25 and

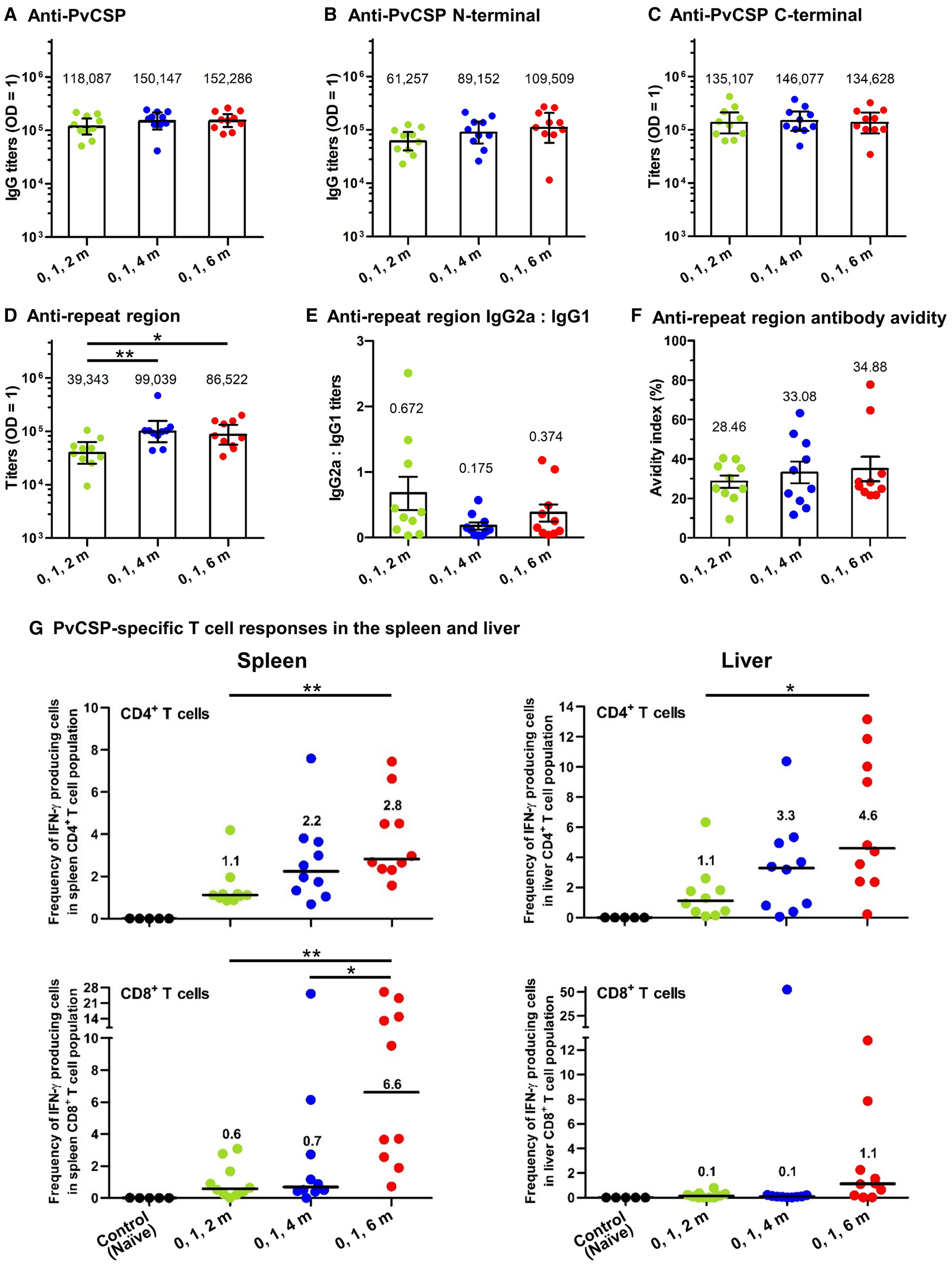

(legend on next page)

Pfs230D1), have shown promising results in preclinical studies.[22,25,26] Recently, our team reported the first use of the mRNA-LNP vaccine platform to target the *P. vivax* transmission-blocking antigen Pvs25, demonstrating its ability to elicit potent functional antibodies.[27] Until now, most efforts to develop CSP-based vaccines against *P. vivax* have concentrated on recombinant proteins, peptides and virus-like particles.[28] This study applies the mRNA-LNP platform to the development of a PvCSP vaccine, evaluating its immunogenicity and protective efficacy. Our data reveal that the PvCSP mRNA-LNP vaccine elicits a highly immunogenic response, generating robust antibody and T cell responses in both the liver and spleen of immunized mice. Importantly, the vaccine provides strong protective efficacy in a transgenic *P. berghei* sporozoite challenge model using CD-1 outbred mice.

CSP, the major surface membrane protein on sporozoites, is targeted by this vaccine to prevent pre-erythrocytic *P. vivax* infection. Neutralizing antibodies block sporozoite liver infection, while T cell responses eliminate sporozoite-infected liver cells. Thus, an effective vaccine, such as the PvCSP vaccine, must aim to induce both neutralizing antibodies and liver T cell responses.

In this study, we designed two nucleoside-modified mRNA-LNPs targeting PvCSP (VK210). While the mRNA construct with the wild-type SP and GPI anchor (SP^+ GPI^+) exhibited lower *in vitro* protein expression, it effectively promoted cell-surface expression of PvCSP on HEK 293T cells and mouse BMDCs. This construct elicited a stronger antibody response compared with the construct containing the wild-type SP but lacking the GPI anchor (SP^+ GPI^-) in mouse immunization studies. The localization of the protein on the cell surface relies on the presence of a GPI anchor at the C-terminal.[29] Our findings support recent observations that mRNA-LNP vaccines encoding either Pvs25 or Pfs25, which also include SP and GPI anchors, generate a superior antibody response compared with those without GPI anchors.[26,27] However, the exact mechanisms behind this enhanced response are still not understood.

DCs are well known for their role in priming T cells by presenting processed antigens through major histocompatibility complex (MHC) class I and II molecules.[30] Some studies have shown that DCs can present native antigens to B cells, leading to antibody production.[31–33] It has been suggested that membrane-associated antigens are more effective at stimulating B cell activation than soluble antigens, as they facilitate receptor cross-linking and antigen capture.[34–36] Thus, we can hypothesize that, after vaccination, antigen-presenting DCs at the injection site take up the PvCSP mRNA (SP^+ GPI^+)-LNP vaccine and present the cell-surface-expressed PvCSP to B cells, potentially enhancing antibody production. Further investigation should focus on effectively targeting DCs with the mRNA-LNP vaccine and understanding how membrane-associated antigens on DCs facilitate B cell activation. This insight is vital for enhancing vaccine efficacy and designing future mRNA-LNP vaccines.

The immune response to the PvCSP mRNA-LNP vaccine could vary depending on the route of administration. Both the IM and ID routes induced a dose-dependent antibody response, with ID administration inducing a significantly stronger response against both the PvCSP and the repeat region at a dose of 10 μg. While IM vaccination is widely used for most vaccines, ID vaccination, such as for the US Food and Drug Administration-approved influenza vaccine, has been shown to offer a dose-sparing effect.[37] The abundance of antigen-presenting Langerhans cells and tissue DCs in the dermis may account for the heightened immune response.[38] Further studies are needed to explore ID vaccination with PvCSP mRNA-LNP vaccine as a strategy to achieve dose-sparing effects without compromising protective efficacy.

In addition to inducing a low antibody response, i.v. administration of the PvCSP mRNA-LNP vaccine also resulted in negligible T cell responses in both the liver and spleen (Figure S3). Our observations differ from prior research showing strong antibody and T cell responses after i.v. administration of mRNA vaccines.[39,40] It is important to note, however, that these previous vaccines used different delivery systems and adjuvants in comparison with the PvCSP mRNA-LNP vaccine.

Several studies have shown that the number of mRNA vaccine doses and the timing of administration are crucial for generating effective protective immunity.[22,41–43] Immunogenicity studies indicate that a delayed third dose regimen administered IM 3 or 5 months after the second dose resulted in significantly higher antibody response specific to the repeat region and stronger T cell responses compared with monthly administrations. Similarly, efficacy studies found that the PvCSP mRNA-LNP vaccine given in a delayed regimen at 0, 1, and 6 months achieved a 90% protection rate, compared with 80% when administered at 0, 1, and 4 months. In contrast, monthly administration at 0, 1, and 2 months resulted in a lower efficacy of

Figure 4. Antibody and T cell responses induced by a three-dose IM vaccine regimen of the PvCSP mRNA-LNP vaccine

Mice ($n = 10$ per group) were IM immunized with three doses of the PvCSP mRNA-LNP vaccine (30 μg/dose) at 0, 1, and 2 months; 0, 1, and 4 months; or 0, 1, and 6 months. Serum antibody responses specific to the PvCSP (A), the N-terminal region (B), the C-terminal region (C), and the repeat region (D) were assessed 2 weeks after administration of the third vaccine dose. Each dot represents one animal. Data in the bar graphs in (A–D) are shown as geometric mean titer (GMTs) and 95% confidence intervals. Statistical significance was determined by one-way ANOVA with Tukey's post test on log-transformed values. The ratio of anti-repeat region IgG2a to IgG1 (E) and the avidity of anti-repeat region antibodies (F) were evaluated. Data in the bar graphs in (E) and (F) are shown as mean ± SEM. $CD4^+$ and $CD8^+$ T cell responses in the spleen and liver were measured for IFN-γ production using ICS assay 4 weeks after administration of the last vaccine dose (G). Spleen and liver tissues of naive mice were used as controls ($n = 5$). Each dot represents one animal, and horizontal lines indicate the medians.

Statistical significance was determined by Kruskal-Wallis test, applying the Bonferroni correction. $*p < 0.05$, $**p < 0.01$.

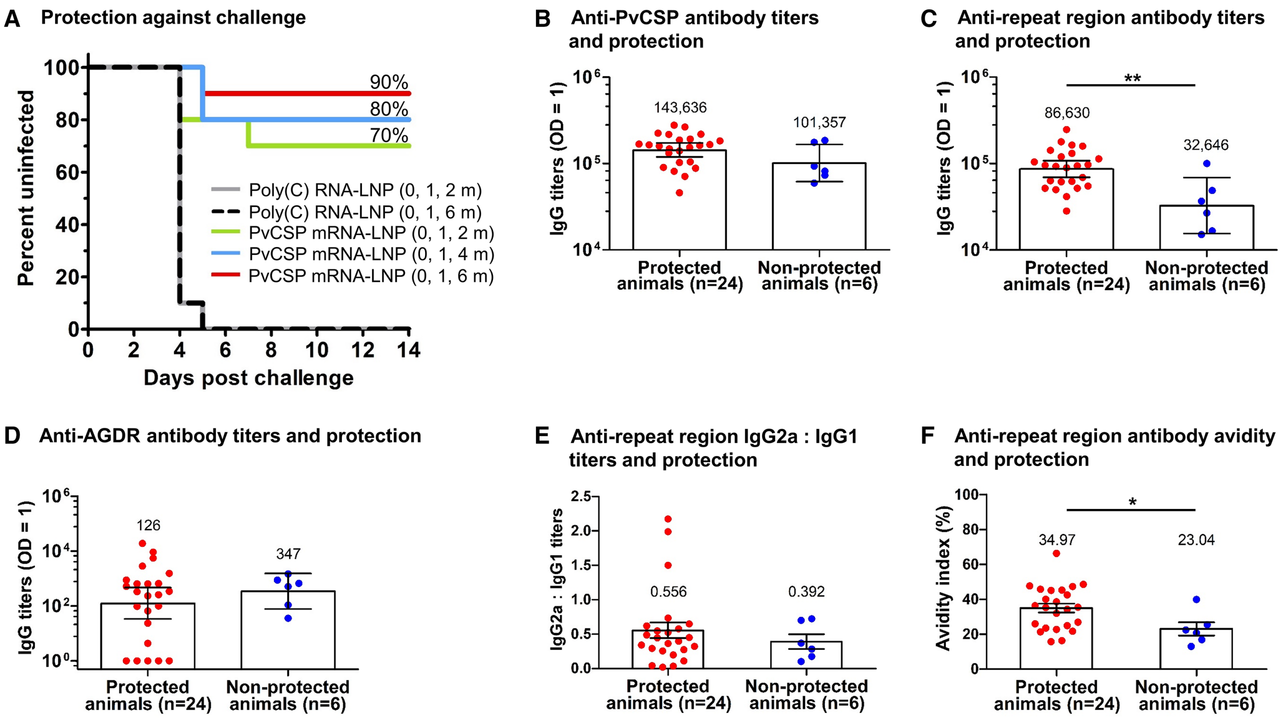

Figure 5. Protection against sporozoite challenge by the PvCSP mRNA-LNP vaccine and its correlation with antibody response

Mice (n = 10 per group) were IM immunized with three doses of the PvCSP-LNP vaccine (30 μg/dose) or control poly(C) RNA-LNP using different vaccination schedules. Three weeks after the final vaccination, animals were challenged with 2,000 transgenic sporozoites administered i.v. Kaplan-Meier survival curves show the percentage of uninfected animals up to day 14, and protective efficacy was calculated (A). The association between protection and the levels of antibodies specific to the PvCSP (B), the repeat region (C), and AGDR peptide (D), as well as the ratio of anti-repeat region IgG2a to IgG1 (E) and the avidity of anti-repeat region IgG antibodies (F), was evaluated. Each dot represents one animal. Data in the bar graphs in (B–D) are shown as GMT and 95% confidence intervals. In (B–D), statistical significance was determined by a Mann-Whitney U test on log-transformed values. $^{**}p < 0.01$. Data in the bar graphs in panels E and F are shown as mean ± SEM. In (E and F), statistical significance was determined by a Mann-Whitney U test. $^{*}p < 0.05$.

70%. The differences in protection outcomes observed in this study are not attributable to the age of the animals at the time of challenge. Mice in the 0-, 1-, and 6-month poly(C) RNA control group were 4 months older at the time of challenge than those in the 0-, 1-, and 2-month control group, yet both control groups developed 100% parasitemia within 5 days. Our findings align with a recent study showing that administering a PvDBPII protein vaccine formulated with Matrix-M adjuvant on a delayed third dose schedule at 0, 1, and 14 months induced high levels of protective antibodies and reduced *P. vivax* blood stage parasite multiplication by 51% in a CHMI study.[44] In contrast, a vaccination schedule at 0, 1, and 2 months did not affect parasite growth.

The exact mechanism responsible for the increased protective efficacy observed with the administration of a delayed third dose of PvCSP mRNA-LNP vaccine in this study remains unclear. The protection provided by the PvCSP mRNA-LNP vaccine was associated with the levels of antibodies against the repeat region. These results reinforce earlier studies that found a correlation between higher antibody titers to the CSP repeat region of *P. vivax* and protection in *Aotus* monkeys immunized with a VMP001 vaccine adjuvanted with a TLR9 agonist.[45] In addition to the levels of anti-repeat region antibodies, we also observed that their avidity was associated with protection. Overall, our findings are consistent with a recent study demonstrating that clinical protection against *P. falciparum* in CHMI studies using the RTS,S vaccine is linked to the magnitude of antibodies (IgG1) directed at the NANP repeat region of PfCSP and their binding avidity.[46]

The tetramer AGDR sequence within the repeat region has been reported to be a target of protective antibodies.[23,24] A vaccine, designed to elicit a strong anti-AGDR response, demonstrated a protective capability.[47] However, in our study, animals immunized with PvCSP mRNA-LNP vaccine developed only a modest anti-AGDR response, and there was no observed association between AGDR antibody titers and protection.

The direct association of T cell responses with protection could not be evaluated in the sporozoite challenge experiment. However, a separated immunogenicity study with a delayed regimen, administered at 0, 1, and 6 months, showed an increase in IFN-γ^+ T cell responses in the spleen and liver, potentially contributing to protective

immunity against liver-stage infection. Previous studies have documented the role of liver-resident and circulating memory $CD8^+$ T cells in clearing liver stage malaria.[40,48] This suggests that PvCSP-specific $CD8^+$ T cells generated by the PvCSP mRNA-LNP vaccine, both in the liver and spleen, may contribute to protection against liver stage infection. The specific role of $CD4^+$ T cell responses in protecting against liver stage infection has been poorly investigated.[49–51] Interestingly, IFN-γ has been reported to inhibit liver stage infection in human and mouse liver cells through the induction of nitric oxide *in vitro*.[52] This inhibition does not impact the sporozoite invasion process or the early stages of infection, but significantly suppressed later liver stage development.[53] Therefore, IFN-γ, secreted by PvCSP-specific $CD4^+$ T cells in the liver and spleen, may inhibit liver-stage parasite development.

It is anticipated that both the magnitude and the avidity of anti-repeat region antibodies, as well as T cell responses in the liver and spleen, contribute to the protective efficacy of the PvCSP mRNA-LNP vaccine. In this study, a relatively high dose (30 μg) of the PvCSP mRNA-LNP vaccine was used and demonstrated strong protection. Based on these promising results, future studies should investigate whether lower doses can achieve comparable immunogenicity and protection. Additional research is needed to identify protective epitopes within the PvCSP repeat region and other domains. Moreover, exploring the potential enhancement of the neutralizing antibody response against subdominant protective epitopes through a delayed third dose regimen is crucial for advancing vaccine development targeting the PvCSP.

The two major circulating *P. vivax* variants, VK210 and VK247, differ primarily in the amino acid sequence of the central repeat region, which is a key target of vaccine-induced protective antibodies. Although our mRNA-LNP vaccine encoding PvCSP (VK210) elicits robust T cell responses, likely directed against epitopes known to be located in the conserved N- and C-terminal regions,[54–56] these responses alone may not confer cross-protection against the VK247 variant. Therefore, further studies are needed to evaluate a construct representing the VK247 vaccine and to explore the development of a bivalent mRNA vaccine encoding PvCSP from both VK210 and VK247 variants, as well as a chimeric mRNA vaccine incorporating the repeat regions of both variants, to achieve broad and effective protection against *P. vivax* infection.

In conclusion, our study highlights the robust immune response induced by the nucleoside-modified PvCSP mRNA-LNP vaccine, which contains the wild-type SP and GPI anchor, including a strong antibody and T cell response in the liver and spleen. Importantly, the vaccine demonstrated significant protection against sporozoite challenge in mice when administered in a three-dose regimen, administered at 0, 1, and 6 months. These findings highlight the crucial importance of vaccine design and timing of vaccination in achieving effective immunity. Overall, our research underscores the potential of the mRNA-LNP platform in developing vaccines to combat *P. vivax* malaria effectively.

MATERIALS AND METHODS

mRNA design and production

Two mRNA-encoding PvCSP constructs were designed based on the gene sequence of the PvCSP (VK210) from Salvador I strain (NCBI Reference Sequence XP_001613068.1). Construct 1 contained the wild-type SP (MKNFILLAVSSILLVDLFPT) and GPI anchor (IFNVVSNSLGLVILLVLALFN), whereas construct 2 contained the wild-type SP, but lacked the GPI anchor. mRNAs were made as described.[57] Briefly, *in vitro* transcription was performed with the codon-optimized DNA constructs with optimized 5′ and 3′ UTRs and poly(A) tail using the MEGAscript T7 Transcription kit (Life Technologies, Carlsbad, CA, USA). To generate modified mRNAs, uridine-5′-triphosphate was replaced with m1Ψ-5′-triphosphate (TriLink BioTechnologies, San Diego, CA, USA). mRNA capping was performed along with transcription through the addition of a trinucleotide cap1 analog, CleanCap (TriLink), and mRNA was purified with cellulose-based purification as described.

LNP encapsulation

Cellulose-purified mRNAs and poly(C) RNA (Sigma Aldrich, St. Louis, MO, USA) were encapsulated in LNPs using a self-assembly process where an aqueous solution of mRNAs at a pH of 4.0 is rapidly mixed with a solution of lipids dissolved in ethanol. LNPs used in this study consisted of a mixture of the ionizable cationic lipid (pKa in the range of 6.0–6.5, proprietary to Acuitas Therapeutics, Vancouver, BC, Canada)/phosphatidylcholine/cholesterol/polyethylene glycol-lipid (50:10:38.5:1.5 mol/mol) as described in the patent application WO 2017/004143. The average hydrodynamic diameter was ∼80 nM with a polydispersity index of 0.02–0.06 as measured by dynamic light scattering using a Zetasizer Nano ZS (Malvern Instruments Ltd, Worcestershire, UK) and an encapsulation efficiency of ∼95% was achieved as determined using a Ribogreen assay (Thermo Fisher Scientific, Waltham, MA, USA).

Cells and cell transfections

HEK 293T cells were cultured in DMEM containing 2 mM L-glutamine (Gibco, Grand Island, NY, USA) and 10% heat-inactivated fetal bovine serum (FBS) (Gibco). Immature mouse BMDCs were prepared as previously described.[58] Briefly, bone marrow was harvested from the femurs and tibias of CD-1 mice. The cell suspension in RPMI medium (Gibco) was filtered through a 70-μm cell strainer (BD Falcon, Durham, NC, USA) to remove bone fragments, and red blood cells were lysed using lysis buffer (BD Biosciences, San Jose, CA, USA). The bone marrow cells were then centrifuged and suspended in complete RPMI medium: RPMI 1640 supplemented with glutamine, penicillin, streptomycin, 2-mercaptoethanol, and 10% heat-inactivated FBS. Bone marrow cells were cultured at a density of 2×10^6 cells per well in 3 mL of complete RPMI medium supplemented with 20 ng/mL of mouse granulocyte-macrophage colony-stimulating factor (GM-CSF) (R&D Systems, Minneapolis, MN, USA) in six-well tissue culture plates. On days 3 and 6, one-half of the medium was removed and replaced with fresh, prewarmed medium containing GM-CSF.

HEK 293T cells were seeded at 4 × 10^5 cells in 1 mL per well in 12-well plates. After overnight culture, cells were then used for transfection. Bone marrow cells were cultured for 7 days. Non-adherent and loosely adherent cells were gently harvested and then were seeded at 1 × 10^6 cells in 0.5 mL per well in 12-well plates. These cells were then used for transfection. Approximately 80% of the day 7 harvested bone marrow cells were DCs, as they were positive for CD11c and MHC-II molecules.

Each well of HEK 293T cells and BMDCs was transfected with 2 μg of mRNA mixed with 4 μL of Lipofectamine 2000 (Life Technologies) in the presence of reduced-serum medium Opti-MEM (Gibco). HEK 293T cells and BMDCs treated with Lipofectamine 2000 were used as controls. After 4 h, transfected HEK 293T cells were washed and then replaced with complete DMEM, whereas transfected BMDCs were washed and then replaced with complete RPMI containing 20 ng/mL of mouse GM-CSF. Culture supernatants were collected 18 h after transfection, and cells were lysed with 0.5 mL of RIPA buffer (Thermo Fisher Scientific) containing a protease inhibitor (Thermo Fisher Scientific) for 30 min on ice.

Cell-surface expression of PvCSP

HEK 293T cells and BMDCs were harvested 15–18 h after transfection, and the cell-surface expression of PvCSP was assessed using flow cytometry. Both transfected and non-transfected HEK 293T cells in 100 μL of PBS were stained with 10 μL of a monoclonal antibody specific to PvCSP (VK210) (BEI Resources, Manassas, VA, USA; cat. No. MRA-1028K) for 30 min, washed twice, and then incubated with an Alexa Fluor 488-conjugated goat anti-mouse IgG antibody (Thermo Fisher Scientific, Eugene, OR, USA). For BMDCs, following the PvCSP staining, the cells were additionally stained with anti-mouse CD11c APC and anti-mouse MHC class II (I-A/I-E) phycoerythrin/Cyanine7 antibodies (BioLegend, San Diego, CA, USA). The stained cells were analyzed for PvCSP expression on HEK 293 cells and CD11c$^+$ MHC II$^+$ BMDCs using a six -color flow cytometer (BD FACSCanto, Becton Dickinson, Mountain View, CA, USA). A portion of the stained cells was cytospun onto slides for observation under a fluorescent microscope (Olympus, Tokyo, Japan).

ELISA

To detect PvCSP expression, secreted PvCSP (VK210) protein in supernatants and in cell lysates were assessed by ELISA kit (BEI Resources). Briefly, 96-well ELISA plates (Nunc maxiSorp, Thermo Fisher Scientific, Roskilde, Denmark) were coated with 50 μL of *P. vivax* (VK210) capture monoclonal antibody (2 μg/mL in PBS) per well and incubated for 1 h at room temperature. The wells were blocked with 200 μL of 0.5% w/v casein in PBS for 1 h. After washing with PBS containing 0.05% Tween 20, samples (supernatants or cell lysates) with 2-fold dilutions were added to each well (50 μL) and incubated for another 2 h. Subsequently, 50 μL of horseradish peroxidase (HRP)-conjugated monoclonal antibody specific to PvCSP (VK210) (1 μg/mL in blocking buffer) was added to each well, and then incubated for another 1 h. The plates were washed three times and 100 μL of ABTS substrate (2,2′-azino-di- (3-ethylbenzothiazoline-6-sulphonic acid and H_2O_2) (KPL, Gaithersburg, MD, USA) was added per well. After incubation for 30 min in the dark, the optical density (OD) was measured at 405 nm using SPECTRAmax Plus plate reader (Molecular Devices, San Jose, CA, USA).

For the detection of antibody responses, mouse serum samples were collected and frozen in liquid nitrogen until use. Antibody responses against PvCSP and different regions were assessed by ELISA. Briefly, 96-well ELISA plates (Nunc MaxiSorp, Thermo Fisher Scientific) were coated with 0.1 μg/well of either recombinant PvCSP (VK210), the repeat region (GDRAAGQPAGDRADGQPA ×2), the N-terminal region, the C-terminal region, or the peptide AGDR ×8 in PBS and incubated in a humidity chamber at 4°C overnight. The plates were then washed using a wash buffer of PBS with 0.1% Tween 20 (Sigma-Aldrich). All wells were filled with blocking buffer composed of 2% BSA in PBS for 1 h at room temperature. The diluent for all sera was 0.4% BSA in washing buffer. Serum samples with 2-fold dilutions, including controls, were incubated on coated, blocked plates for 2 h at room temperature. The HRP-conjugated goat anti-mouse IgG (Abcam, Waltham, MA, USA) was diluted in at 1:10,000 and 100 μL added to all wells and incubated for 1 h at room temperature. Plates were then washed and the ABTS substrate (KPL) for development was added to each well and incubated for 15 min. At the end of this incubation, the reaction was stopped using 5% sodium dodecyl sulfate stop solution (KPL). The plates were read at 405 nm on a SPECTRAmax plate reader (Molecular Devices), and serial dilution curves were analyzed using SoftMax Pro 5.2 software. Titer was defined as the dilution factor needed to give an OD of 1.0 in our assay.

Recombinant proteins and peptides

Purified recombinant proteins PvCSP (VK210), along with the N-terminal and C-terminal regions of PvCSP expressed in *E. coli*, were provided by Dr. Anjali Yadava from the WRAIR. The repeat region (GDRAAGQPAGDRADGQPA ×2) and the tetramer (AGDR ×8) were synthesized by GenScript (Hong Kong, China).

Mouse immunization

CD-1 outbred mice (female animals aged 6–9 weeks) were used throughout the study for testing immunogenicity and protective efficacy. Male mice were not used due to their aggressiveness, resulting in frequent fighting when housed together in the same cage.

In the first experiment assessing the immunogenicity of two constructs of PvCSP mRNA-LNP vaccines, groups of mice (n = 7) were immunized IM with 30 μg per dose at 0 and 4 weeks. Serum samples were collected 1 week after the second vaccine dose and assessed for antibody response. The spleen and liver were collected 29–41 days after the second vaccine dose for measurement of the memory T cell response.

In the second experiment evaluating the effects of administration routes, groups of mice (n = 5) received the PvCSP mRNA-LNP

vaccine at different doses administered IM (10 and 30 μg), ID (10 and 30 μg), or i.v. (10 and 30 μg) at 0 and 4 weeks. Serum samples were collected 1 week after the second vaccine dose and assessed for antibody response. To assess the memory T cell response, the spleen and liver were collected 29–41 days after the second vaccine dose.

In the third experiment assessing the effects of vaccination schedules, groups of mice (n = 10) received IM immunization with 30 μg/dose of PvCSP mRNA-LNP using a three-dose regimen administered at either 0, 1, and 2 months; 0, 1, and 4 months; or 0, 1, and 6 months. Serum samples were collected 2 weeks after the third vaccine dose and assessed for antibody response. The spleen and liver were collected 4 weeks after the third vaccine dose for the measurement of memory T cell response.

In the final experiment evaluating the protective efficacy of the PvCSP mRNA-LNP vaccine, groups of mice (n = 10) were IM vaccinated with 30 μg/dose at either 0, 1, and 2 months; 0, 1, and 4 months; or 0, 1, and 6 months. Two separate control groups were used: one received poly(C) RNA (30 μg per dose) at months 0, 1, and 2, and the other at months 0, 1, and 6.

Seven days after the final vaccine dose, serum samples were collected to assess the antibody response. Twenty-two days after the last vaccine dose, mice were challenged with transgenic *P. berghei* sporozoite expressing PvCSP (VK210).

IgG subclass measurement

IgG subclass against the repeat-region of PvCSP (VK210) were assessed by ELISA. Briefly, ELISA plates (Nunc maxiSorp, Thermo Fisher Scientific) were coated with 0.1 μg/well with the repeat region (GDRAAGQPAGDRADGQPA ×2) as described earlier. Serum samples with 2-fold dilutions were added into separated plates for IgG1 and IgG2a. The HRP-conjugated goat anti-mouse IgG1 or IgG2a (Abcam) at 1:10,000 dilution were added (100 μL/well) and developed by ABTS substrate (KPL) for 15 min and reaction was stopped using 5% sodium dodecyl sulfate stop solution (KPL). The plates were read at 405 nm and the titer was defined as described earlier.

IFA

The IFA was used to assess antibody responses against whole *P. vivax* sporozoites (VK210) isolated from *Anopheles dirus* mosquitoes fed with blood collected from *P. vivax*-infected patients (provided by Dr. Jetsumon Sattabongkot) or transgenic *P. berghei* expressing PvCSP (VK210). To prepare sporozoite slides, separate multiwell slides (Electron Microscopy Sciences, Hatfield, PA, USA) were coated with sporozoites (10^3–10^4/well), air dried, and fixed with methanol. The sporozoite slides were blocked with 1% BSA in PBS for 20 min at room temperature. Serum samples at different dilutions in PBS-BSA, were added to the wells, and the slides were incubated in a humidified chamber for 1 h at room temperature. The slides were washed with PBS, and Alexa Fluor 488-conjugated goat anti-mouse IgG antibody (Thermo Fisher Scientific) at a dilution of 1:250 was added for 1 h at room temperature. Slides were washed, and mounted in ProLong Glass Antifade Mountant (Invitrogen, Carlsbad, CA, USA), and viewed with fluorescent microscope (Olympus).

Avidity ELISA

A sodium thiocyanate ELISA was used to determine anti-repeat region antibody avidity. ELISA plates (Nunc maxiSorp, Thermo Fisher Scientific) coated with the repeat region (GDRAAGQPAGDRAD GQPA ×2) for 2 h were blocked with 2% BSA in PBS for 1 h at room temperature. Serum samples were added and two identical plates were generated. After washing, one identical plate was incubated with 100 μL of PBS, while the second identical plate was incubated with 100 μL of 1.5 M sodium thiocyanate (Sigma-Aldrich) in PBS (chaotrope solution) for 20 min at room temperature. ELISA plates were washed and then precede for second antibody reaction and substrate development as earlier described. The avidity index was calculated with this equation: (OD 1 titer in chaotrope solution/OD 1 titer in PBS) × 100.

Preparation of mononuclear immune cells and intracellular cytokine staining

Spleens were homogenized between the frosted ends of two slides, while livers were chopped into small pieces and were mashed through 70 μm cell strainers (BD Falcon). Single-cell suspensions from spleen and liver were centrifuged using Histopaque-1077 to obtain mononuclear immune cells. Contaminated red blood cells were removed by treating with ammonium chloride-based lysing solution (BD Biosciences). Purified mononuclear immune cells were used for assessment of PvCSP-specific T cell responses using intracellular cytokine staining (ICS). Due to our previous observations that frozen mononuclear immune cells from the liver exhibited low cell viability, we consistently used freshly isolated cells from the liver for ICS.

Mononuclear immune cells were resuspended in media consisting of RPMI1640 supplemented with antibiotic/antimycotic, L-glutamine, sodium pyruvate, MEM non-essential amino acids, 2-mercaptoethanol, and 5% FBS (all from Gibco). Overall, cell viability was greater than 95%, as assessed by propidium iodide (Invitrogen, Eugene, OR, USA) staining and analyzed using 6-color flow cytometry (BD FACSCanto, Becton Dickinson). Cells (1×10^6 cells in 200 μL of medium) were stimulated with PvCSP (VK210) peptide (92 15-mer peptide overlapping by 11 amino acids) at a final concentration of each peptide of 1.5 μg/mL. Three samples per tissue were used: (1) mononuclear immune cells cultured with medium served as an unstimulated control (background); (2) mononuclear immune cells stimulated with PMA (50 ng/mL) and ionomycin (1 μg/mL) served as a positive control; and (3) mononuclear immune cells stimulated with PvCSP (VK210) peptide served as an experimental sample. After the initial 2 h of stimulation, GolgiPlug (diluted 1:1,000 from the stock; BD Biosciences) was added to inhibit cytokine secretion and cultured overnight. After 18–20 h of incubation, cells were washed and stained with fluorescence-conjugated monoclonal antibodies specific to CD3 (clone 145-2C11), CD4 (clone RM4-5), and CD8 (clone 53-6.7) for

30 min and then washed. The stained cells were treated with fixation/permeabilization solution (BD Bioscience) and then stain with fluorescence-conjugated monoclonal antibodies specific to IFN-γ (clone XMG1.2) and intertleukin-2 (clone JES6-5H4). All the monoclonal antibodies used were obtained from BioLegend. Finally, the stained cells were analyzed by six-color flow cytometry (BD FACSCanto, Becton Dickinson). The values of unstimulated controls (background) were deducted from PvCSP-specific T cell responses before being reported.

Sporozoite challenge

Transgenic *P. berghei* parasite expressing PvCSP (VK210) was obtained from Dr. Shigeto Yoshida[59] (Kanazawa University, Kanazawa, Ishikawa, Japan) under a cooperative research and development agreement. Sporozoites were harvested from the salivary glands of infected *Anopheles stephensi* mosquitoes and were suspended in PBS with 10% heat-inactivated fetal calf serum (FCS). The sporozoite preparations were maintained on ice and used within 2 h. CD-1 outbred mice were challenged with i.v. administration of 2,000 sporozoites in 100 μL of PBS containing 5% FCS (lateral caudal veins of the tail) per mouse. Mice were assessed for blood stage infection at day 4 post challenge through day 14 using Giemsa-stained thick and thin blood smears. Blood parasitemia was measured using microscopy. Mice that tested negative for parasitemia up to day 14 after the challenge were considered to be sterilely protected. Kaplan-Meier survival curves were used to represent protective efficacy to a sporozoite challenge. Vaccine efficacy was calculated using the formula: Efficacy = [1 − [(number of infected animals $(I)_{\text{vaccine}}$/total number of animals $(n)_{\text{vaccine}}$) ÷ (number of infected animals $(I)_{\text{control}}$/total number of animals $(n)_{\text{control}}$)]] × 100).

Statistical analysis

Data were analyzed using SPSS 12.0 for Windows (SPSS, Chicago, IL). Antibody titers were subjected to a logarithmic transformation before testing of differences. Statistical significance for two group comparisons were determined using a paired t test or Mann-Whitney U test. Statistical significance for multiple group comparisons were determined using one-way ANOVA with Tukey's post-test or Kruskal-Wallis tests with Bonferroni correction. A p value of <0.05 was considered significant.

DATA AVAILABILITY

The datasets generated and/or analyzed during the current study are available from the corresponding authors on reasonable request.

ACKNOWLEDGMENTS

All animal research was conducted under an IACUC-approved animal use protocol in an AAALAC International-accredited facility with a Public Health Services Animal Welfare Assurance and in compliance with the Animal Welfare Act and other federal statutes and regulations relating to laboratory animals. We thank all the members of the study teams from the Veterinary Medicine and Entomology departments at AFRIMS for their dedication and excellent work. We also thank Dr. Jetsumon Sattabongkot from the Faculty of Tropical Medicine, Mahidol University, Bangkok, Thailand, for providing *P. vivax* sporozoites. This work was supported by the Military Infectious Diseases Research program (MIDRP) and by a grant from NIAID, NIH (grant 1U01AI155361 awarded to J.A.). The Pardi laboratory is supported by the National Institute of Allergy and Infectious Diseases (NIAID, R01AI146101 and R01AI153064). Material has been reviewed by the Walter Reed Army Institute of Research. There is no objection to its presentation and/or publication. The opinions or assertions contained herein are the private views of the author, and are not to be construed as official, or as reflecting true views of the Department of the Army or the Department of Defense.

AUTHOR CONTRIBUTIONS

Conceptualization: S.P., N.P., A.Y., A.L., U.K., N.W., and B.A.V. Performing experiments: A.L., U.K., K.Y., R.I., R.U., and H.M. mRNA design: N.P. Supervision of the production of the LNPs: Y.K.T. Providing transgenic parasites: S.Y. Data analysis: A.L., U.K., K.Y., and S.P. Funding acquisition: S.P., J.A., N.P., and D.W. Writing – original draft: A. L. and U.K. Writing – review and editing: S.P. and N.P.

DECLARATION OF INTERESTS

A.Y. is named on a patent describing the application of a synthetic *P. vivax* circumsporozoite protein as a diagnostic reagent, for antibody production, and as a vaccine protective against infection with any strain of *P. vivax*. D.W. and N.P. are named on a patent describing the use of modified mRNA in lipid nanoparticles as a vaccine platform. N.P. served on the mRNA strategic advisory board of Sanofi Pasteur in 2022 and Pfizer in 2023–2024, is a member of the scientific advisory boards of AldexChem and BioNet, and has consulted for Vaccine Company, and Pasture Biosciences. Y.K.T. is an employee of Acuitas Therapeutics.

SUPPLEMENTAL INFORMATION

Supplemental information can be found online at https://doi.org/10.1016/j.omtn.2025.102645.

REFERENCES

1. Manzoni, G., Try, R., Guintran, J.O., Christiansen-Jucht, C., Jacoby, E., Sovannaroth, S., Zhang, Z., Banouvong, V., Shortus, M.S., Reyburn, R., et al. (2024). Progress towards malaria elimination in the Greater Mekong Subregion: perspectives from the World Health Organization. Malar. J. *23*, 64. https://doi.org/10.1186/s12936-024-04851-z.
2. WHO (2023). World Malaria Report 2023 (World Health Organization). https://www.who.int/teams/global-malaria-programme/reports/world-malaria-report-2023.
3. Phillips, M.A., Burrows, J.N., Manyando, C., van Huijsduijnen, R.H., Van Voorhis, W.C., and Wells, T.N.C. (2017). Malaria. Nat. Rev. Dis. Primers *3*, 17050. https://doi.org/10.1038/nrdp.2017.50.
4. Battle, K.E., Gething, P.W., Elyazar, I.R.F., Moyes, C.L., Sinka, M.E., Howes, R.E., Guerra, C.A., Price, R.N., Baird, K.J., and Hay, S.I. (2012). The global public health significance of Plasmodium vivax. Adv. Parasitol. *80*, 1–111. https://doi.org/10.1016/B978-0-12-397900-1.00001-3.
5. Menkin-Smith, L., and Winders, W.T. (2024). Plasmodium vivax Malaria. In StatPearls. Treasure Island (FL).
6. Howes, R.E., Battle, K.E., Mendis, K.N., Smith, D.L., Cibulskis, R.E., Baird, J.K., and Hay, S.I. (2016). Global Epidemiology of Plasmodium vivax. Am. J. Trop. Med. Hyg. *95*, 15–34. https://doi.org/10.4269/ajtmh.16-0141.
7. Markus, M.B. (2011). Malaria: origin of the term "hypnozoite". J. Hist. Biol. *44*, 781–786. https://doi.org/10.1007/s10739-010-9239-3.
8. Baird, K. (2015). Origins and implications of neglect of G6PD deficiency and primaquine toxicity in Plasmodium vivax malaria. Pathog. Glob. Health *109*, 93–106. https://doi.org/10.1179/2047773215Y.0000000016.
9. Nkhoma, E.T., Poole, C., Vannappagari, V., Hall, S.A., and Beutler, E. (2009). The global prevalence of glucose-6-phosphate dehydrogenase deficiency: a systematic review and meta-analysis. Blood Cells Mol. Dis. *42*, 267–278. https://doi.org/10.1016/j.bcmd.2008.12.005.
10. Watson, J., Taylor, W.R.J., Bancone, G., Chu, C.S., Jittamala, P., and White, N.J. (2018). Implications of current therapeutic restrictions for primaquine and tafenoquine in the radical cure of vivax malaria. PLoS Negl. Trop. Dis. *12*, e0006440. https://doi.org/10.1371/journal.pntd.0006440.
11. Zavala, F. (2022). RTS,S: the first malaria vaccine. J. Clin. Investig. *132*, e156588. https://doi.org/10.1172/JCI156588.

12. WHO (2023). WHO recommends R21/Matrix-M vaccine for malaria prevention in updated advice on immunization. 2 October 2023, News release, Geneva. https://www.who.int/publications/m/item/r21-matrix-m-malaria-vaccine–evidence-to-recommendations-framework–2023.

13. Bennett, J.W., Yadava, A., Tosh, D., Sattabongkot, J., Komisar, J., Ware, L.A., McCarthy, W.F., Cowden, J.J., Regules, J., Spring, M.D., et al. (2016). Phase 1/2a Trial of Plasmodium vivax Malaria Vaccine Candidate VMP001/AS01B in Malaria-Naive Adults: Safety, Immunogenicity, and Efficacy. PLoS Negl. Trop. Dis. *10*, e0004423. https://doi.org/10.1371/journal.pntd.0004423.

14. Arevalo-Herrera, M., Gaitan, X., Larmat-Delgado, M., Caicedo, M.A., Herrera, S.M., Henao-Giraldo, J., Castellanos, A., Devaud, J.C., Pannatier, A., Onate, J., et al. (2022). Randomized clinical trial to assess the protective efficacy of a Plasmodium vivax CS synthetic vaccine. Nat. Commun. *13*, 1603. https://doi.org/10.1038/s41467-022-29226-3.

15. Baden, L.R., El Sahly, H.M., Essink, B., Kotloff, K., Frey, S., Novak, R., Diemert, D., Spector, S.A., Rouphael, N., Creech, C.B., et al. (2021). Efficacy and Safety of the mRNA-1273 SARS-CoV-2 Vaccine. N. Engl. J. Med. *384*, 403–416. https://doi.org/10.1056/NEJMoa2035389.

16. Polack, F.P., Thomas, S.J., Kitchin, N., Absalon, J., Gurtman, A., Lockhart, S., Perez, J.L., Pérez Marc, G., Moreira, E.D., Zerbini, C., et al. (2020). Safety and Efficacy of the BNT162b2 mRNA Covid-19 Vaccine. N. Engl. J. Med. *383*, 2603–2615. https://doi.org/10.1056/NEJMoa2034577.

17. Hogan, M.J., and Pardi, N. (2022). mRNA Vaccines in the COVID-19 Pandemic and Beyond. Annu. Rev. Med. *73*, 17–39. https://doi.org/10.1146/annurev-med-042420-112725.

18. Rice, M.C., and O'Brien, S.J. (1980). Genetic variance of laboratory outbred Swiss mice. Nature *283*, 157–161. https://doi.org/10.1038/283157a0.

19. Martin, M.D., Danahy, D.B., Hartwig, S.M., Harty, J.T., and Badovinac, V.P. (2017). Revealing the Complexity in CD8 T Cell Responses to Infection in Inbred C57B/6 versus Outbred Swiss Mice. Front. Immunol. *8*, 1527. https://doi.org/10.3389/fimmu.2017.01527.

20. Herzog, C. (2014). Influence of parenteral administration routes and additional factors on vaccine safety and immunogenicity: a review of recent literature. Expert Rev. Vaccines *13*, 399–415. https://doi.org/10.1586/14760584.2014.883285.

21. Rosenbaum, P., Tchitchek, N., Joly, C., Rodriguez Pozo, A., Stimmer, L., Langlois, S., Hocini, H., Gosse, L., Pejoski, D., Cosma, A., et al. (2021). Vaccine Inoculation Route Modulates Early Immunity and Consequently Antigen-Specific Immune Response. Front. Immunol. *12*, 645210. https://doi.org/10.3389/fimmu.2021.645210.

22. Mallory, K.L., Taylor, J.A., Zou, X., Waghela, I.N., Schneider, C.G., Sibilo, M.Q., Punde, N.M., Perazzo, L.C., Savransky, T., Sedegah, M., et al. (2021). Messenger RNA expressing PfCSP induces functional, protective immune responses against malaria in mice. NPJ Vaccines *6*, 84. https://doi.org/10.1038/s41541-021-00345-0.

23. Charoenvit, Y., Collins, W.E., Jones, T.R., Millet, P., Yuan, L., Campbell, G.H., Beaudoin, R.L., Broderson, J.R., and Hoffman, S.L. (1991). Inability of malaria vaccine to induce antibodies to a protective epitope within its sequence. Science *251*, 668–671. https://doi.org/10.1126/science.1704150.

24. Jones, T.R., Yuan, L.F., Marwoto, H.A., Gordon, D.M., Wirtz, R.A., and Hoffman, S. L. (1992). Low immunogenicity of a Plasmodium vivax circumsporozoite protein epitope bound by a protective monoclonal antibody. Am. J. Trop. Med. Hyg. *47*, 837–843. https://doi.org/10.4269/ajtmh.1992.47.837.

25. Hayashi, C.T.H., Cao, Y., Clark, L.C., Tripathi, A.K., Zavala, F., Dwivedi, G., Knox, J., Alameh, M.G., Lin, P.J.C., Tam, Y.K., et al. (2022). mRNA-LNP expressing PfCSP and Pfs25 vaccine candidates targeting infection and transmission of Plasmodium falciparum. NPJ Vaccines *7*, 155. https://doi.org/10.1038/s41541-022-00577-8.

26. Scaria, P.V., Roth, N., Schwendt, K., Muratova, O.V., Alani, N., Lambert, L.E., Barnafo, E.K., Rowe, C.G., Zaidi, I.U., Rausch, K.M., et al. (2024). mRNA vaccines expressing malaria transmission-blocking antigens Pfs25 and Pfs230D1 induce a functional immune response. NPJ Vaccines *9*, 9. https://doi.org/10.1038/s41541-023-00783-y.

27. Kunkeaw, N., Nguitragool, W., Takashima, E., Kangwanrangsan, N., Muramatsu, H., Tachibana, M., Ishino, T., Lin, P.J.C., Tam, Y.K., Pichyangkul, S., et al. (2023). A Pvs25 mRNA vaccine induces complete and durable transmission-blocking immunity to Plasmodium vivax. NPJ Vaccines *8*, 187. https://doi.org/10.1038/s41541-023-00786-9.

28. da Veiga, G.T.S., Moriggi, M.R., Vettorazzi, J.F., Müller-Santos, M., and Albrecht, L. (2022). Plasmodium vivax vaccine: What is the best way to go? Front. Immunol. *13*, 910236. https://doi.org/10.3389/fimmu.2022.910236.

29. Saha, S., Anilkumar, A.A., and Mayor, S. (2016). GPI-anchored protein organization and dynamics at the cell surface. J. Lipid Res. *57*, 159–175. https://doi.org/10.1194/jlr.R062885.

30. Guermonprez, P., Valladeau, J., Zitvogel, L., Théry, C., and Amigorena, S. (2002). Antigen presentation and T cell stimulation by dendritic cells. Annu. Rev. Immunol. *20*, 621–667. https://doi.org/10.1146/annurev.immunol.20.100301.064828.

31. Bergtold, A., Desai, D.D., Gavhane, A., and Clynes, R. (2005). Cell surface recycling of internalized antigen permits dendritic cell priming of B cells. Immunity *23*, 503–514. https://doi.org/10.1016/j.immuni.2005.09.013.

32. Qi, H., Egen, J.G., Huang, A.Y.C., and Germain, R.N. (2006). Extrafollicular activation of lymph node B cells by antigen-bearing dendritic cells. Science *312*, 1672–1676. https://doi.org/10.1126/science.1125703.

33. Steiner, T.M., Heath, W.R., and Caminschi, I. (2022). The unexpected contribution of conventional type 1 dendritic cells in driving antibody responses. Eur. J. Immunol. *52*, 189–196. https://doi.org/10.1002/eji.202149658.

34. Batista, F.D., Iber, D., and Neuberger, M.S. (2001). B cells acquire antigen from target cells after synapse formation. Nature *411*, 489–494. https://doi.org/10.1038/35078099.

35. Carrasco, Y.R., and Batista, F.D. (2006). B-cell activation by membrane-bound antigens is facilitated by the interaction of VLA-4 with VCAM-1. EMBO J. *25*, 889–899. https://doi.org/10.1038/sj.emboj.7600944.

36. Heath, W.R., Kato, Y., Steiner, T.M., and Caminschi, I. (2019). Antigen presentation by dendritic cells for B cell activation. Curr. Opin. Immunol. *58*, 44–52. https://doi.org/10.1016/j.coi.2019.04.003.

37. Atmar, R.L., Patel, S.M., and Keitel, W.A. (2010). Intanza((R)): a new intradermal vaccine for seasonal influenza. Expert Rev. Vaccines *9*, 1399–1409. https://doi.org/10.1586/erv.10.134.

38. Romani, N., Flacher, V., Tripp, C.H., Sparber, F., Ebner, S., and Stoitzner, P. (2012). Targeting skin dendritic cells to improve intradermal vaccination. Curr. Top. Microbiol. Immunol. *351*, 113–138. https://doi.org/10.1007/82_2010_118.

39. Haabeth, O.A.W., Lohmeyer, J.J.K., Sallets, A., Blake, T.R., Sagiv-Barfi, I., Czerwinski, D.K., McCarthy, B., Powell, A.E., Wender, P.A., Waymouth, R.M., and Levy, R. (2021). An mRNA SARS-CoV-2 Vaccine Employing Charge-Altering Releasable Transporters with a TLR-9 Agonist Induces Neutralizing Antibodies and T Cell Memory. ACS Cent. Sci. *7*, 1191–1204. https://doi.org/10.1021/acscentsci.1c00361.

40. Ganley, M., Holz, L.E., Minnell, J.J., de Menezes, M.N., Burn, O.K., Poa, K.C.Y., Draper, S.L., English, K., Chan, S.T.S., Anderson, R.J., et al. (2023). mRNA vaccine against malaria tailored for liver-resident memory T cells. Nat. Immunol. *24*, 1487–1498. https://doi.org/10.1038/s41590-023-01562-6.

41. Falsey, A.R., Frenck, R.W., Jr., Walsh, E.E., Kitchin, N., Absalon, J., Gurtman, A., Lockhart, S., Bailey, R., Swanson, K.A., Xu, X., et al. (2021). SARS-CoV-2 Neutralization with BNT162b2 Vaccine Dose 3. N. Engl. J. Med. *385*, 1627–1629. https://doi.org/10.1056/NEJMc2113468.

42. Wu, K., Choi, A., Koch, M., Elbashir, S., Ma, L., Lee, D., Woods, A., Henry, C., Palandjian, C., Hill, A., et al. (2021). Variant SARS-CoV-2 mRNA vaccines confer broad neutralization as primary or booster series in mice. Vaccine *39*, 7394–7400. https://doi.org/10.1016/j.vaccine.2021.11.001.

43. Pajon, R., Doria-Rose, N.A., Shen, X., Schmidt, S.D., O'Dell, S., McDanal, C., Feng, W., Tong, J., Eaton, A., Maglinao, M., et al. (2022). SARS-CoV-2 Omicron Variant Neutralization after mRNA-1273 Booster Vaccination. N. Engl. J. Med. *386*, 1088–1091. https://doi.org/10.1056/NEJMc2119912.

44. Hou, M.M., Barrett, J.R., Themistocleous, Y., Rawlinson, T.A., Diouf, A., Martinez, F.J., Nielsen, C.M., Lias, A.M., King, L.D.W., Edwards, N.J., et al. (2023). Vaccination with Plasmodium vivax Duffy-binding protein inhibits parasite growth during controlled human malaria infection. Sci. Transl. Med. *15*, eadf1782. https://doi.org/10.1126/scitranslmed.adf1782.

45. Yadava, A., Hall, C.E., Sullivan, J.S., Nace, D., Williams, T., Collins, W.E., Ockenhouse, C.F., and Barnwell, J.W. (2014). Protective efficacy of a Plasmodium vivax circumsporozoite protein-based vaccine in Aotus nancymaae is associated with antibodies to the repeat region. PLoS Negl. Trop. Dis. *8*, e3268. https://doi.org/10.1371/journal.pntd.0003268.

46. Spreng, R.L., Seaton, K.E., Lin, L., Hilliard, S., Horn, G.Q., Abraha, M., Deal, A.W., Li, K., Carnacchi, A.J., Feeney, E., et al. (2024). Identification of RTS,S/AS01 vaccine-induced humoral biomarkers predictive of protection against controlled human malaria infection. JCI Insight *9*, e178801. https://doi.org/10.1172/jci.insight.178801.

47. Atcheson, E., and Reyes-Sandoval, A. (2020). Protective efficacy of peptides from Plasmodium vivax circumsporozoite protein. Vaccine *38*, 4346–4354. https://doi.org/10.1016/j.vaccine.2020.03.063.

48. Lefebvre, M.N., Surette, F.A., Anthony, S.M., Vijay, R., Jensen, I.J., Pewe, L.L., Hancox, L.S., Van Braeckel-Budimir, N., van de Wall, S., Urban, S.L., et al. (2021). Expeditious recruitment of circulating memory CD8 T cells to the liver facilitates control of malaria. Cell Rep. *37*, 109956. https://doi.org/10.1016/j.celrep.2021.109956.

49. Oliveira, G.A., Kumar, K.A., Calvo-Calle, J.M., Othoro, C., Altszuler, D., Nussenzweig, V., and Nardin, E.H. (2008). Class II-restricted protective immunity induced by malaria sporozoites. Infect. Immun. *76*, 1200–1206. https://doi.org/10.1128/IAI.00566-07.

50. Renia, L., Grillot, D., Marussig, M., Corradin, G., Miltgen, F., Lambert, P.H., Mazier, D., and Del Giudice, G. (1993). Effector functions of circumsporozoite peptide-primed CD4+ T cell clones against Plasmodium yoelii liver stages. J. Immunol. *150*, 1471–1478.

51. Charoenvit, Y., Majam, V.F., Corradin, G., Sacci, J.B., Jr., Wang, R., Doolan, D.L., Jones, T.R., Abot, E., Patarroyo, M.E., Guzman, F., and Hoffman, S.L. (1999). CD4(+) T-cell- and gamma interferon-dependent protection against murine malaria by immunization with linear synthetic peptides from a Plasmodium yoelii 17-kilodalton hepatocyte erythrocyte protein. Infect. Immun. *67*, 5604–5614. https://doi.org/10.1128/IAI.67.11.5604-5614.1999.

52. Mellouk, S., Hoffman, S.L., Liu, Z.Z., de la Vega, P., Billiar, T.R., and Nussler, A.K. (1994). Nitric oxide-mediated antiplasmodial activity in human and murine hepatocytes induced by gamma interferon and the parasite itself: enhancement by exogenous tetrahydrobiopterin. Infect. Immun. *62*, 4043–4046. https://doi.org/10.1128/iai.62.9.4043-4046.1994.

53. Patel, H., Minkah, N.K., Kumar, S., Zanghi, G., Schepis, A., Goswami, D., Armstrong, J., Abatiyow, B.A., Betz, W., Reynolds, L., et al. (2024). Malaria blood stage infection suppresses liver stage infection via host-induced interferons but not hepcidin. Nat. Commun. *15*, 2104. https://doi.org/10.1038/s41467-024-46270-3.

54. Nardin, E., Clavijo, P., Mons, B., van Belkum, A., Ponnudurai, T., and Nussenzweig, R.S. (1991). T cell epitopes of the circumsporozoite protein of Plasmodium vivax. Recognition by lymphocytes of a sporozoite-immunized chimpanzee. J. Immunol. *146*, 1674–1678.

55. Herrera, S., Escobar, P., de Plata, C., Avila, G.I., Corradin, G., and Herrera, M.A. (1992). Human recognition of T cell epitopes on the Plasmodium vivax circumsporozoite protein. J. Immunol. *148*, 3986–3990.

56. Lumsden, J.M., Pichyangkul, S., Srichairatanakul, U., Yongvanitchit, K., Limsalakpetch, A., Nurmukhambetova, S., Klein, J., Bertholet, S., Vedvick, T.S., Reed, S.G., et al. (2011). Evaluation of the safety and immunogenicity in rhesus monkeys of a recombinant malaria vaccine for Plasmodium vivax with a synthetic Toll-like receptor 4 agonist formulated in an emulsion. Infect. Immun. *79*, 3492–3500. https://doi.org/10.1128/IAI.05257-11.

57. Vadovics, M., Muramatsu, H., Sárközy, A., and Pardi, N. (2024). Production and Evaluation of Nucleoside-Modified mRNA Vaccines for Infectious Diseases. Methods Mol. Biol. *2786*, 167–181. https://doi.org/10.1007/978-1-0716-3770-8_7.

58. Dong, Y., Arif, A.A., Poon, G.F.T., Hardman, B., Dosanjh, M., and Johnson, P. (2016). Generation and Identification of GM-CSF Derived Alveolar-like Macrophages and Dendritic Cells From Mouse Bone Marrow. J. Vis. Exp. *112*, e54194. https://doi.org/10.3791/54194.

59. Mizutani, M., Iyori, M., Blagborough, A.M., Fukumoto, S., Funatsu, T., Sinden, R.E., and Yoshida, S. (2014). Baculovirus-vectored multistage Plasmodium vivax vaccine induces both protective and transmission-blocking immunities against transgenic rodent malaria parasites. Infect. Immun. *82*, 4348–4357. https://doi.org/10.1128/IAI.02040-14.

OMTN, Volume *36*

Supplemental information

mRNA-LNP vaccine encoding the *Plasmodium vivax* circumsporozoite protein is highly immunogenic and confers protection in mice

Amporn Limsalakpetch, Utaiwan Kum-Arb, Kosol Yongvanitchit, Rawiwan Im-Erbsin, Ratawan Ubalee, Norman Waters, Brian A. Vesely, Hiromi Muramatsu, Drew Weissman, Ying K. Tam, Shigeto Yoshida, John Adams, Anjali Yadava, Norbert Pardi, and Sathit Pichyangkul

SUPPLEMENTAL MATERIAL

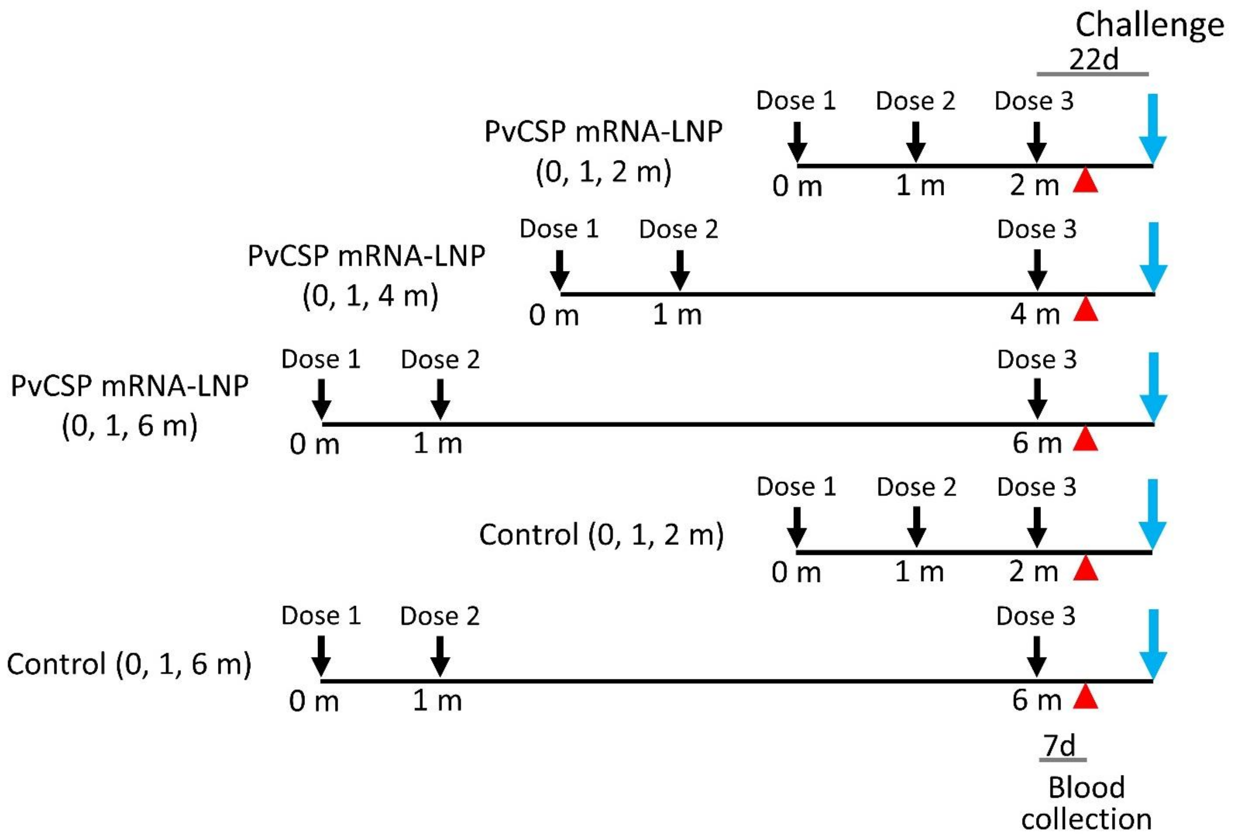

Figure S1. Vaccination regimen for testing the protective efficacy of the PvCSP mRNA-LNP vaccine

Mice (n = 10 per group) were IM immunized with 3 doses of the PvCSP mRNA-LNP vaccine (30 µg) at either 0, 1, and 2 months; 0, 1, and 4 months; or 0, 1, and 6 months. Two separate control groups received poly(C) RNA-LNP (30 µg per dose) at 0, 1, and 2 months, or at 0, 1, and 6 months. Serum samples were collected seven days after the final vaccine dose to measure antibody responses. All mice were challenged with transgenic sporozoites twenty two days after the last vaccine dose.

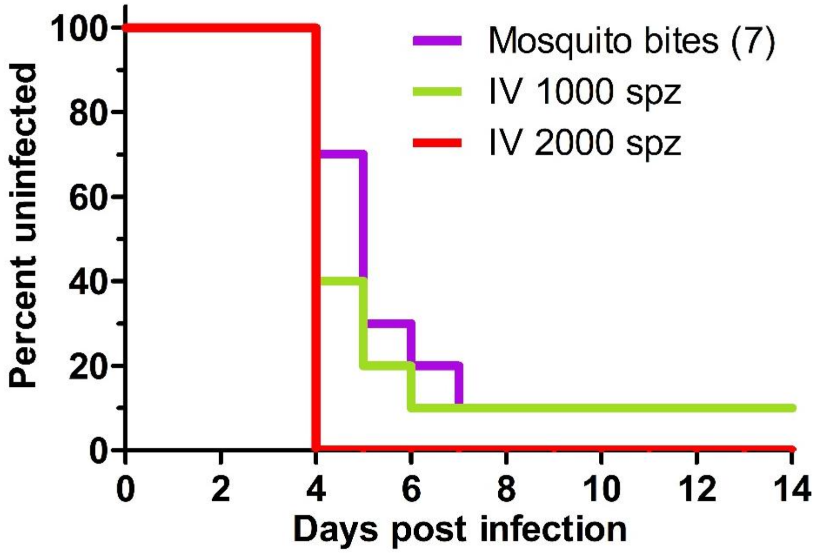

Figure S2. Transgenic P. berghei sporozoites expressing PvCSP (VK210) effectively establish a blood stage parasitemia in CD-1 outbred mice

In the study, mice (n=10 per group) were exposed to seven mosquito bites, or administered 1,000 or 2,000 sporozoites intravenously (IV). Administration of 2,000 sporozoites resulted in a 100% incidence of blood stage infection.

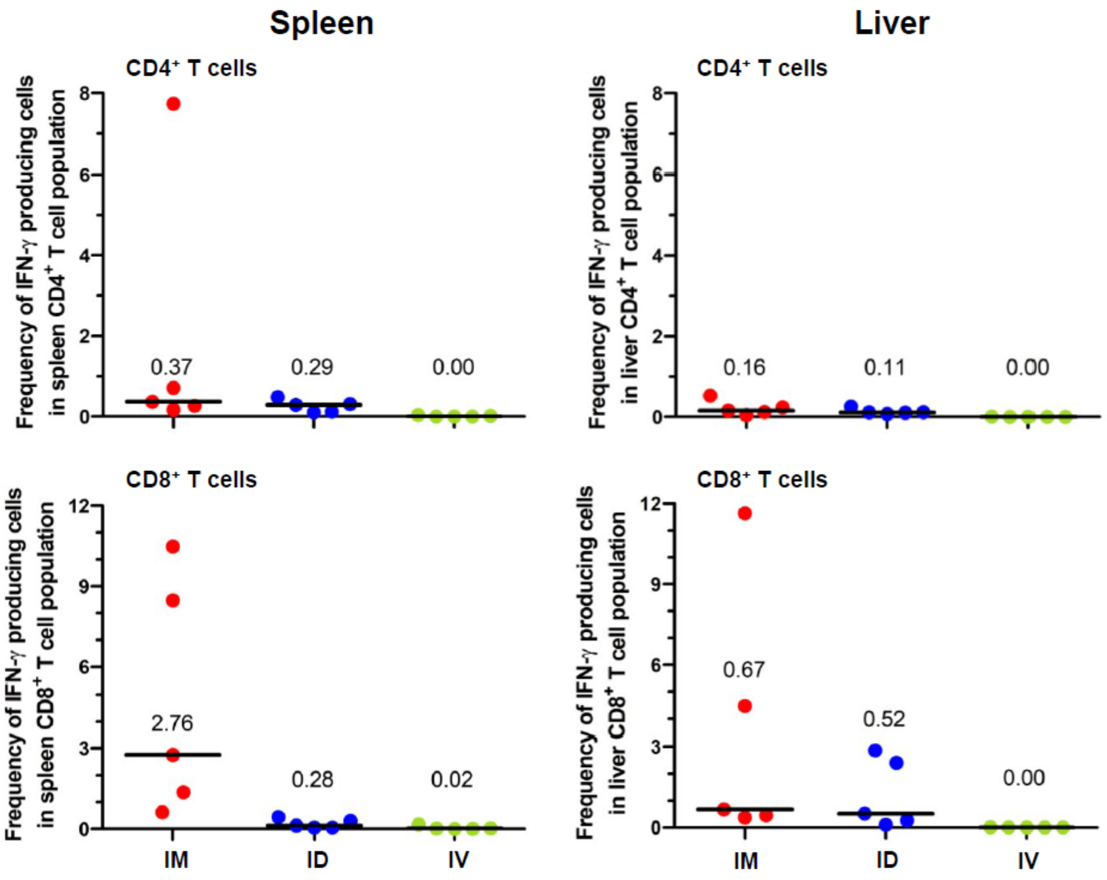

Figure S3. IV administration of the PvCSP mRNA-LNP vaccine induces a minimal T cell response compared to the IM and ID administration routes

Mice (n=5 per group) were immunized with the PvCSP mRNA-LNP vaccine (30 µg/dose) via IM, ID, or IV routes at 0 and 4 weeks. CD4^{+} *and CD8*$^{+}$ *memory T cell responses in the spleen and liver were measured for cytokine production 29-41 days after the final vaccination using an ICS assay. Each dot represents one animal, and horizontal lines indicate the medians.*
